# Comparative Efficacy of Brolucizumab and Aflibercept in Polypoidal Choroidal Vasculopathy: A Systematic Review and Meta-Analysis

**DOI:** 10.7759/cureus.77073

**Published:** 2025-01-07

**Authors:** Manar H Allehyani, Abdullah K Alsaeedi, Reem O Alqthmi, Raghad E Saleh, Rawan S Alsamli, Hussam A Almalki, Abdulrahman F Alshehri, Saja A Felimban, Gufran J Kambiji, Mohammad I Almatrafi, Basant Othman

**Affiliations:** 1 General Surgery, King Abdulaziz Hospital, Makkah, SAU; 2 Ophthalmology, Alnoor Specialist Hospital, Makkah, SAU; 3 Neurology, King Abdulaziz Hospital, Makkah, SAU; 4 Medicine and Surgery, Umm Al-Qura University, Makkah, SAU; 5 Medicine, Umm Al-Qura University, Makkah, SAU

**Keywords:** aflibercept, brolucizumab, comparative effectiveness, meta-analysis, polypoidal choroidal vasculopathy

## Abstract

Polypoidal choroidal vasculopathy (PCV) represents a distinct subtype of neovascular age-related macular degeneration (nAMD). PCV is currently managed using intravitreal anti-vascular endothelial growth factor (VEGF) agents such as brolucizumab and aflibercept. This meta-analysis compares the effectiveness of brolucizumab and aflibercept in PCV patients. We systematically searched four electronic databases to identify eligible studies. Data extraction and pooling were performed utilizing the mean difference (MD) or rate ratio (RR) through the generic inverse variance method, with significance determined by a p-value < 0.05 between intervention subgroups. The generic inverse variance analysis method was applied with the employment of the random-effect model when data were heterogeneous.

We retrieved 44 studies, 35 were included in the meta-analysis. The analysis compared the efficacy of aflibercept and brolucizumab in patients with nAMD over 3-6 months and 12 months. For best-corrected visual acuity (BCVA), the MD between aflibercept and brolucizumab were -0.11 versus -0.06 at 3-6 months and -0.11 versus -0.04 at 12 months, with no substantial differences (p = 0.58 and p = 0.08, respectively). Regarding polypoidal regression, RR after aflibercept use was 53% versus 70% for brolucizumab at 3-6 months and 47% versus 61% at 12 months, with no significant differences (p = 0.19 and p = 0.31, respectively). In terms of central retinal thickness (CRT), the MDs for aflibercept versus brolucizumab were -129.03 versus -143.93 at 3-6 months and -129.72 versus -145.32 at 12 months, without significant differences (p = 0.62 for both). For central choroidal thickness (CCT) and central foveal thickness (CFT), no significant differences were found between the two interventions at either time point. However, for central macular thickness, brolucizumab demonstrated superiority over aflibercept at 12 months (MD = -119.29 versus -215.00, p < 0.0001). In conclusion, our meta-analysis comparing aflibercept and brolucizumab in PCV revealed no significant differences in BCVA, polypoidal regression, CRT, CCT, and CFT at 6 or 12 months. Overall, both drugs demonstrated comparable efficacy in managing PCV.

## Introduction and background

Polypoidal choroidal vasculopathy (PCV) is a type of neovascular age-related macular degeneration (nAMD), with polypoidal dilatations and a branching vascular network (BVN). Typically, these features are situated between Bruch's membrane and the retinal pigment epithelium (RPE) [1]. Recurrent sub-retinal hemorrhages (SRHs) and fluid accumulation frequently accompany this condition. Left untreated, it can result in irreversible vision loss [1,2]. Indocyanine green angiography (ICGA) is the gold standard for detecting aneurysmal dilations, characteristic of polypoidal lesions in PCV [2,3].

PCV is prevalent among Asian populations, with reported prevalence rates ranging from 22% to 62% [3,4]. Estimates of PCV prevalence among Caucasian participants have been reported at 7%-10% [5]. However, PCV cases are likely underestimated in these populations due to the infrequent use of ICGA [6]. More recent investigations conducted in Caucasian populations utilizing ICGA have indicated higher rates of PCV, ranging between 20% and 31% [7,8].

PCV is managed using intravitreal anti-vascular endothelial growth factor (VEGF) agents alone or combined with photodynamic therapy (PDT) [9]. Nevertheless, a significant challenge persists due to the high treatment and monitoring visit burden, particularly in underserviced areas [10]. There is a critical unmet need for effective treatments capable of prolonging intervals between injections while preserving visual gains.

Intravitreal brolucizumab injections (IVB) are a single-chain antibody fragment with high anti-VEGF affinity. Its low molecular weight enables the administration of a higher drug dose per injection compared to other anti-VEGF agents. This property holds the potential for enhanced tissue penetration and prolonged duration of action [11]. Many phase 3 clinical trials, specifically the HAWK and HARRIER studies, demonstrated that IVB effectively improved and maintained visual acuity for 96 weeks. This efficacy was found to be non-inferior to that observed with intravitreal aflibercept injections (IVA) administered every eight weeks [12]. Furthermore, it has been reported that IVB exhibited superior efficacy in controlling macular fluid, intraretinal fluid, sub-retinal fluid (SRF), and sub-RPE fluid, compared to IVA [12]. 

Previous investigations have documented the therapeutic efficacy of IVB in patients diagnosed with PCV, yielding promising outcomes [13,14]. However, there is currently a lack of sufficient reports examining the differences between IVB and other anti-VEGF agents within PCV cohorts. This meta-analysis, the first in this context, aims to identify and analyze the differences in the efficacy of IVB and IVA in treating PCV.

## Review

Methods

The investigation conducted in our research adhered strictly to the guidelines set forth by the Preferred Reporting Items for Systematic Reviews and Meta-analyses (PRISMA) and meticulously followed the recommendations delineated in the Cochrane Handbook for Systematic Reviews of Interventions [15,16].

Literature Search

We searched the following databases: Scopus, Web of Science, PubMed, and Cochrane, to procure pertinent literature. The search encompassed literature from its inception until March 2024. Furthermore, a manual examination of reference lists from eligible articles was carried out to ensure comprehensive coverage and to identify any potentially overlooked relevant citations. The search strategy employed a blend of predetermined terms to maximize inclusivity and relevance as follows: (("Polypoidal Choroidal Vasculopathy" OR PCV) AND (Brolucizumab OR Beovu OR RTH-258 OR DLX 1008 OR S01LA06 OR "ESBA1008" OR Aflibercept OR "VEGF Trap-Regeneron" OR "VEGF Trap-Eye" OR EYLEA OR Zaltrap OR "AVE 0005" OR "AVE 005" OR ZIV-aflibercept OR "EYLEA HD" OR "Bay 86-5321" OR "BAY 865321")).

Inclusion Criteria

Two reviewers autonomously screened the pertinent references and assessed their appropriateness according to pre-established criteria. The inclusion criteria encompassed studies meeting the following conditions: (1) confirmation of active PCV utilizing multimodal imaging; (2) incorporation of treatments involving either IVB or IVA; and (3) studies featuring a minimum follow-up period of three months to be included in the analysis; (4) studies featuring any of the following outcomes were included in the analysis-best-corrected visual acuity (BCVA), central retinal thickness (CRT), central macular thickness, central choroidal thickness (CCT), central foveal thickness (CFT), and polypoidal regression.

Several studies were excluded from our analysis due to specific exclusion criteria, including 1) studies involving eyes with a prior history of uveitis, a documented treatment history for PCV, and those presenting with other maculopathy such as exudative age-related macular degeneration (AMD) lacking evidence of polypoidal lesions on ICGA were excluded from the analysis; 2) basic science studies; 3) studies not published in English; 4) and unavailability of full-text.

Data Gathering

The data extraction process entailed employing a data extraction sheet to collect relevant information from each included study systematically. The collected data encompassed various aspects, including the first author and publication year, study location, patient numbers, gender distribution, participant ages, BCVA, logarithm of the minimum angle of resolution (logMAR), treatment regimen, follow-up duration, inclusion criteria, study conclusions, and primary outcomes.

Quality Assessment

The quality assessment of the trials included in the analysis utilized the Cochrane Risk of Bias assessment tool 1 (ROB1) [17]. Each domain, comprising allocation concealment, random sequence generation, blinding of investigators and participants, selective reporting, attrition bias, and other biases, was meticulously assessed to evaluate potential biases within the included studies comprehensively. The quality evaluation of the cohort and case-series studies included in our analysis was evaluated using the National Institutes of Health (NIH) tool [18]. Additionally, we employed the Methodological Index for Non-Randomized Studies (MINORS) Criteria for assessing non-randomized clinical trials. These criteria encompass seven domains, including consecutive patient inclusion, study aim, prospective data collection, evaluation of endpoints, appropriate endpoint selection, follow-up period, and the percentage of loss of follow-up [19]. 

Data Synthesis

All statistical analyses were conducted using appropriate meta-analytic methods. Continuous outcomes were analyzed utilizing the mean difference (MD) and standard error, whereas dichotomous outcomes were assessed using the rate ratio (RR) and corresponding standard error. ubsequently, the significance between subgroups of the two studied interventions was determined when the p-value < 0.05 between the subgroups. The generic inverse variance analysis method was employed for this purpose. Statistical heterogeneity among the studies was evaluated using I-squared (I^2^) and chi-squared (Chi^2^) statistics. A Chi^2^- p-value of less than 0.10 indicates the presence of heterogeneity, while an I^2^ value of ≥ 50% denotes substantial heterogeneity. In cases of substantial heterogeneity, a random-effects model was utilized.

Results

Results of Literature Search

**Figure 1:**
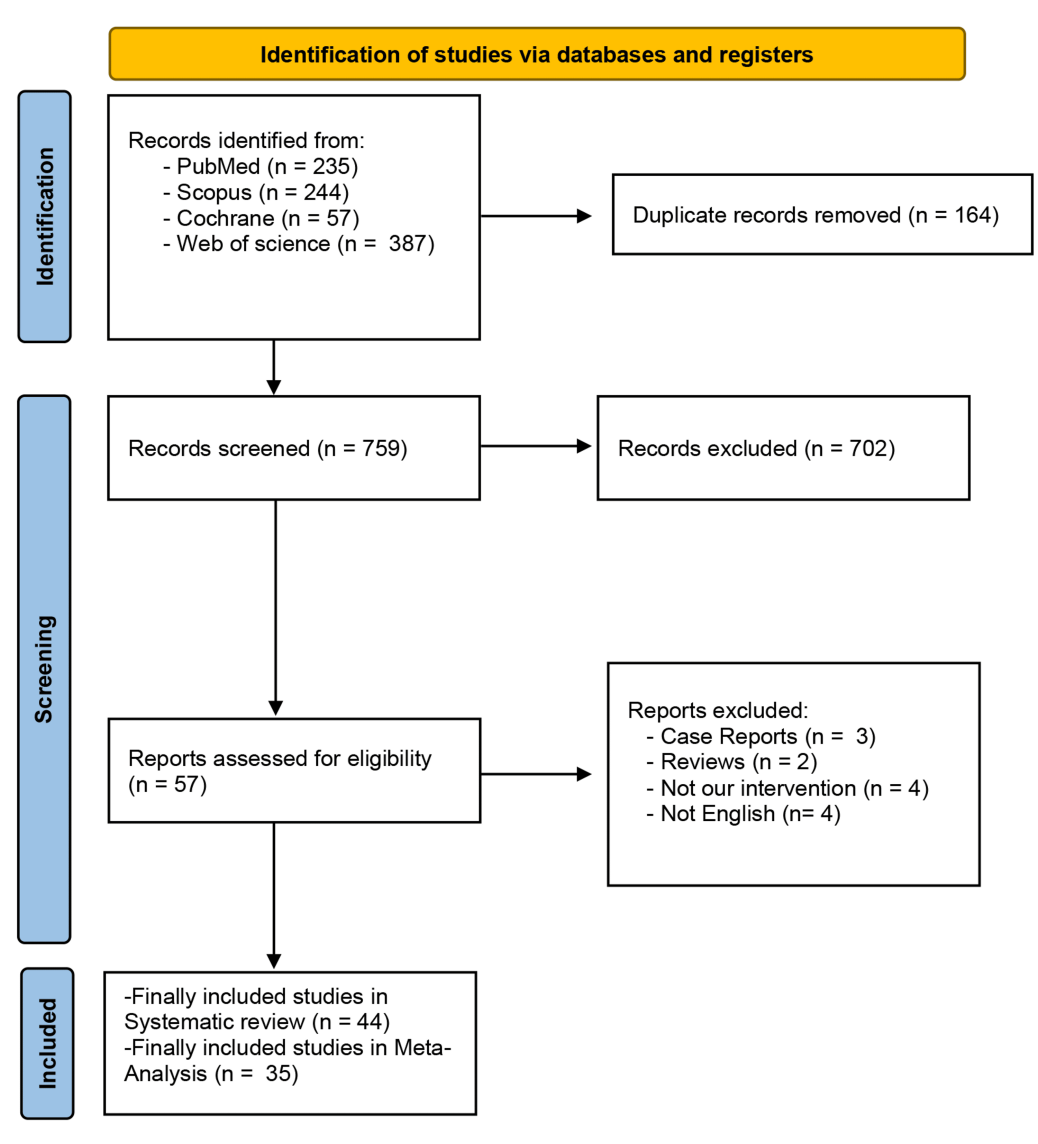
PRISMA flow chart PRISMA: Preferred Reporting Items for Systematic Reviews and Meta-analyses

The primary search across four databases yielded a total of 923 studies. After removing duplicate studies, 759 unique articles remained for further evaluation. Following title and abstract screening, 57 records met the eligibility criteria for full-text screening. Among these, 13 studies were excluded based on predefined criteria. Ultimately, 44 studies were deemed eligible and met the inclusion criteria for our systematic review, with 35 being suitable for meta-analysis [13,20-62]. The PRISMA flow diagram illustrating the study selection process is depicted in (Figure 1).

Characteristics of Included Studies

Our systematic review encompassed 44 studies involving 2028 cases in the IVA group and 349 cases in the IVB group. 29 studies were cohort, 11 were non-randomized controlled trails, three were case series, and one was randomized controlled trails. These studies were conducted across nine countries, with a predominant number performed in Japan and South Korea. Most of the follow-up durations ranged from 3 to 12 months. The primary endpoints for most studies were BCVA, CRT, and polypoidal regression. The baseline summary and characteristics for the included studies are outlined in (Table 1).

**Table 1:** Summary and baseline characteristics of the included studies PCV: Polypoidal choroidal vasculopathy; VEGF: Vascular endothelial growth factor; BCVA: Best corrected visual acuity; ICGA: Indocyanine green angiography; RCT: Randomized control trial; IPCV: Idiopathic polypoidal choroidal vasculopathy; CMT: Central macular thickness; IVA: Intravitreal aflibercept injection; CRT: Central retinal thickness; IVB: Intravitreal brolucizumab injection; CCT: Central choroidal thickness; AMD: Age-related macular degeneration; CSMT: Central subfield mean thickness; IVZ: Intravitreal zip-aflipercipt; CVH: Choroidal vascular hyperpermeability; PED: Pigment epithelial detachment; TAE: Treat-and-extend; OCTA: Optical coherence tomography angiography; PRN: Pro re nata; CNV: Choroidal neovascularization; RPE: Retinal pigment epithelium; PDT: Photodynamic therapy; OCT: Optical coherence tomography; ELM: External limiting membrane; MS: Mean sensitivity; CFT: Central foveal thickness; SRF: Sub-retinal fluid; LogMAR: Logarithm of the minimum angle of resolution; OR: Odds ratio; FA: Fluorescein angiography; SRH: Sub-retinal hemorrhage; EDI: Enhanced depth imaging; BVN: Branching vascular network; ETDRS: Early treatment diabetic retinopathy study; nAMD: Neovascular age-related macular degeneration; wAMD: Wet age-related macular degeneration; MD: Mean difference

N	Study ID	Agent, N	Etiology/study arms, n (%)	Site	Study design	Male, n (%)	Age in years, (mean ± SD)	BCVA, (mean ± SD) LogMAR	Follow-up duration (months)	Treatment regimen	Inclusion criteria	Primary endpoints	Conclusion
1	Cho 2023 [32]	Aflibercept, 40	PCV (100%)	Korea	Retrospective Cohort	29 (72.5%)	67.3±9.3	0.44±0.38	12	IVB (6.0 mg/0.05 mL). IVA (2.0 mg/0.05 mL).	1. Confirmation of active PCV. 2. No previous history of anti-VEGF treatment. 3. Treatment with IVB or IVA (2.0 mg/0.05 mL). 4. Complete a follow-up period of more than 12 months.	The visual and anatomical outcome for PCV after injections of brolucizumab in comparison with aflibercept.	"Intravitreal brolucizumab injections for PCV showed visual improvement comparable to that of aflibercept during the 12-month treatment period. However, brolucizumab was more effective than aflibercept for the regression of polypoidal lesions and caused a greater decrease in CRT and sub-foveal choroidal thickness."
		Brolucizumab, 22				14 (63.6%)	68.7±8.8	0.46±0.40					
2	Fukuda 2021 [35]	Aflibercept, 38	PCV (100%)	Japan	Retrospective Cohort	27 (71.1%)	71.3±10.0	0.30±0.30	3	IVB (6.0 mg/0.05 mL). IVA (2.0 mg/0.05 mL). Plus monthly injections.	1. Treatment-naïve eyes with PCV. 2. Eyes receiving 3-monthly IVA or IVB.	Short-term visual, morphological, and angiographic outcomes for PCV after 3-monthly IVB in comparison with IVA.	"In short-term follow-up, intravitreal injection of 3-monthly brolucizumab was comparable with aflibercept in terms of BCVA and morphological improvement along with higher resolution of polypoidal lesion(s) on ICGA."
		Brolucizumab, 14				10 (71.4%)	74.7±7.3	0.27±0.34					
3	Fukuda 2023 [36]	Aflibercept, 33	PCV (100%)	Japan	Retrospective Cohort	24 (72.7%)	71.6±7.7	0.24±0.25	12	IVB (6.0 mg/0.05 mL). IVA (2.0 mg/0.05 mL).	1. Treatment-naïve eyes. 2. Follow-up duration over 12 months. 3. Treatment regimen of 3-monthly intravitreal injections; thereafter, as-needed additional injections. 4. Available FA/ICGA images at baseline, 3 months, and 12 months.	The visual and anatomical outcome for PCV after IVB in comparison with IVA.	"In treatment-naïve eyes with PCV, the as-needed administration regimen of brolucizumab was comparable to aflibercept in terms of visual and anatomical outcomes, with fewer additional injections during the 12-month follow-up."
		Brolucizumab, 23				19 (82.6%)	74.5±8.3	0.30±0.30					
4	Ogura 2021 [13]	Aflibercept, 39	PCV (100%)	Multicenter	RCT	NA	NA	NA	22	IVB (6 mg). IVA (2 mg).	1. Aged ≥50 years. 2. BCVA between 78 and 23 ETDRS.	The visual acuity and anatomic results of brolucizumab compared with aflibercept treatment in eyes with PCV.	"In Japanese eyes with PCV, brolucizumab q12w/q8w monotherapy resulted in robust and consistent BCVA gains that were comparable to q8w aflibercept dosing. Anatomical outcomes favored brolucizumab over aflibercept, with 76% of brolucizumab participants maintained on q12w dosing after loading to week 48."
		Brolucizumab, 30											
5	Arakawa 2017 [29]	Aflibercept, 22	PCV (100%)	Japan	Non-RCT	15 (68.2%)	62.5±8.8	NA	12	IVA (2 mg/0.05 mL) 3 initial monthly doses, then 4 times every 2 months.	1. Age ≥50 years with active sub-foveal lesions. 2. The presence of polypoidal lesions was identified using ICGA. 3. The presence of dye leakage was observed using FA. 4. The presence of subretinal and/or intraretinal exudative changes observed using OCT.	Evaluation of the efficacy of IVA therapy in treatment-naïve Japanese patients with PCV.	"Japanese patients with treatment-naïve PCV, who were treated with intravitreal aflibercept every 2 months after 3 initial monthly doses, exhibited a significant increase in ETDRS letter scores and a high rate of polyp resolution at 12 months."
6	Azuma 2016 [30]	Aflibercept, 17	PCV (100%)	Japan	Retrospective Cohort	10 (58.8%)	73.8±7.4	0.30±0.29	12	IVA. 3 consecutive doses monthly.	1. Patient enrolled from February 2013 to August 2014 at the outpatient clinic of Tokyo University Hospital. 2. Ranibizumab-resistant PCV.	Angiopathic findings of IVA for ranibizumab-resistant PCV.	"A TAE regimen with intravitreal aflibercept for ranibizumab resistant patients resulted in BVN shrinkage over 1 year."
7	Chakraborty 2022 [31]	Brolucizumab, 21	PCV (100%)	India	Retrospective Cohort	17 (81%)	65.1±9.9	NA	6.5	IVB 6 mg (Pagenax, Novartis, India). Loading dose, 3 months monthly + TAE.	1. All consecutive patients with a diagnosis of PCV at baseline. 2. Treated with IVB 6 mg (Pagenax, Novartis, India).	Efficacy of brolucizumab 6 mg for managing treatment-resistant and treatment‑naïve eyes with PCV.	"Brolucizumab is safe and effective in controlling PCV disease in both treatment-resistant and treatment‑naïve eyes."
8	Chi Moon 2015 [25]	Aflibercept, 32	nAMD, 18 PCV, 14	Korea	Retrospective Chart Review	16 (55.2%)	63.8±11.3	NA	9	IVA (2 mg in 0.05 mL).	1. CNV secondary to AMD or PCV diagnosed with FA. 2. Patient enrolled from July 1, 2013, to June 30, 2014.	Visual and anatomical response of IVA in eyes with nAMD and PCV.	"Aflibercept seems to be effective for improvement and maintenance of BCVA and CMT for nAMD and PCV refractory to anti-VEGF. Switching from aflibercept back to bevacizumab treatment may not be a proper strategy."
9	Choo 2021 [33]	Aflibercept, 25	PCV (100%)	Korea	Case Series	20 (80%)	67.16±8.20	0.53±0.31	12	IVA. Loading phase, once a month for 3 months. Maintenance injections, every 2 months.	1. Patients with confirmed PCV by ICGA. 2. OCT shows SRF or intraretinal fluid. 3. BCVA between 20/200 and 20/20 Patients without a history of treatment with intravitreal injections (ranibizumab, bevacizumab or aflibercept). 4. Patients without a history of treatment with PDT with verteporfin.	1-year outcomes of fixed-dosing aflibercept therapy for the treatment of PCV.	"The fixed-dosing aflibercept regimen is effective for treating patients with PCV and is more effective in treatment-naïve patients than in pre-treated patients."
10	Dabir 2023 [20]	Brolucizumab, 59	nAMD, 48 (80.4%) PCV, 11 (19.6%)	India	Retrospective Chart Review	24 (52.2%)	74.06±5.6	0.60±0.4	-	IVB (6 mg in 0.05 mL).	1. The patient enrolled from January 2021 to June 2022. 2. They had been previously treated with other anti-VEGF agents.	Efficacy and safety profile of brolucizumab in the treatment of nAMD and IPCV.	"Brolucizumab promises a reduced number of injections with longer treatment intervals."
11	Daizumoto 2016 [21]	Aflibercept, 40	PCV (100%)	Japan	Retrospective Cohort	21 (52.5%)	72.9±10.3	0.334±0.371	12	IVA (2.0 mg). Loading dose, 3 consecutive doses monthly. Additional dose, IVAs given as needed.	1. The patient enrolled from October 2013 to November 2015. 2. Patient diagnosed with PCV.	Assessment of the changes of the choroidal structure after IVAs for PCV.	"The binarization of the EDI-OCT images can be used to quantify the activity of PCV and to predict the prognosis after IVA."
12	Farooq 2017 [34]	Aflibercept, 20	PCV (100%)	Georgia	Non-RCT	11 (55%)	68±35.11	NA	12	IVA. Loading dose, 2 mg for 3 months. Mandatory 2 mg every 2 months for 12 months.	1. Age ≥ 18 years. 2. ICGA and FA characteristics consistent with active, leaking neovascular PCV.	Incidence and severity of ocular and systemic adverse events.	"At 1 year, neovascular PCV in a pre-dominantly non-Asian population treated with IVA demonstrated favorable visual, anatomic, and safety outcomes."
13	Fukuda 2020 [22]	Aflibercept, 110	Pachydrusen, 16 (14.5%) No Drusen, 45 (41%) Soft Drusen, 35 (31%) PCV/Scarring, 14 (13.5%)	Japan	Retrospective Cohort	12 (75%) 37 (73.3%) 27 (77.14%) 10 (71.43%)	68.4 69.7 75.0 77.9	0.26±0.32 0.39±0.38 0.35±0.29 0.31±0.35	12	IVA (2.0 mg/0.05 mL). Maintenance, 3 doses monthly.	1. Symptomatic PCV with foveal exudation. 2. Follow-up period equal or more than 12 months. 3. Baseline BCVA from 0.06 to 1.0 in decimal format.	Efficacy of aflibercept for treatment of PCV of the different groups.	"Patients with pachydrusen in fellow eyes were less likely to require additional IVAs following the loading dose and may be ideal candidates for aflibercept monotherapy in their first year."
14	Hara 2016 [37]	Aflibercept, 29	PCV (100%)	Japan	Retrospective Cohort	22 (78.6%)	74±8	0.54±0.48	12	IVA (2.0 mg/0.05 mL). Loading dose of 3-monthly injections. At 3 months, an additional intravitreal dose applied.	1. The patients enrolled from December 2012 to June 2014. 2. Progressing visual symptoms.	Efficacy of aflibercept (2.0 mg) in the treatment-naïve eyes with PCV during 1 year.	"Aflibercept is effective for the eyes with treatment-naive PCV to achieve the resolution of polypoidal lesions. The authors need to carefully observe the eyes after confirming the complete resolution of the polypoidal lesion because of recurrent polyps seen in one-quarter of the study eyes."
15	Hoshino 2024 [38]	Brolucizumab, 46	Signal disappearance, 31 (67.4%) Signal persistence, 15 (32.6%)	Japan	Retrospective Cohort	35 (76%)	73.3±8.0	0.27±0.30 0.16±0.17	12	IVB (6 mg/0.05 ml). Loading phase, 3 monthly. Maintenance phase, TAE regimen with IVB. A minimum of 8 weeks and a maximum of 16 weeks.	1. The patients enrolled from June 2020 to May 2022 at Gunma University Hospital. 2. Completed a 1-year treatment protocol with brolucizumab.	Efficacy of brolucizumab for treatment of PCV.	"PCV cases that showed disappearance of blood flow signals within polypoidal lesions on OCTA after the loading-phase treatment with IVB showed favorable 1-year treatment outcomes. Evaluating blood flow signals within polypoidal lesions by OCTA may allow noninvasive prediction of the treatment outcomes of PCV."
16	Hosokawa 2015 [40]	Aflibercept, 18	PCV (100%)	Japan	Case Series	14 (82.4%)	75.03±19.4	0.414±0.384	6	IVA (2 mg). Loading phase, 3-monthly intervals + 1 additional injection 2 months late.	1. Patients enrolled from 5 February 5, 2013, to June 20, 2013. 2. Diagnosed with PCV.	Efficacy of IVAs for PCV with a focus on the regression of polyps.	"At 6 months, aflibercept monotherapy effectively reduced polyps, retinal exudation and CRT in patients with PCV."
17	Hosokawa 2016 [39]	Aflibercept, 37	PCV (100%)	Japan	Retrospective Cohort	30 (81.1%)	72.4±26.2	0.37±0.37	12	IVA (2 mg). Loading dose, 3 monthly.	Patients enrolled between June 2013 and August 2014.	To evaluate the 1-year treatment outcomes of IVA using a TAE regimen for PCV.	"IVA using a TAE regimen is effective for improving BCVA and CRT in eyes with PCV."
18	Ijiri 2015 [41]	Aflibercept, 33	PCV (100%)	Japan	Case Series	17 (51.5%)	75±8.7	0.40±0.34	14	IVA 2.0 mg. Loading dose, monthly for 3 months.	1. Patients enrolled from December 2012 to August 2013. 2. Presence of elevated orange-red lesions. 3. PCV was diagnosed in all eyes after confirmation of the presence of polypoidal lesions.	Efficacy of aflibercept monotherapy in patients with treatment-naïve PCV.	"Intravitreal aflibercept was well-tolerated in patients with treatment-naïve PCV over the short-term."
19	Inoue 2015 [42]	Aflibercept, 42	Every 8-Week Group, 25 (59.5%) PRN Group, 17 (40.5%)	Japan	Non-RCT	19 (76%) 13 (76.5%)	71.7±7.1 71.1 ± 10.6	0.31±0.08 0.33±0.13	12	IVA (0.05 mL/2.0 mg). Loading doses, 3 initial monthly injections. Maintenance dose, every 2 months injection (every 8-week group) or PRN dosing regimen (PRN group).	1. Patients enrolled from March 2013 to October 2013. 2. Confirmed PCV diagnosis.	Functional and morphologic outcomes of patients with PCV undergoing IVA treatment.	"Among the 2 treatment modalities, IVA was well tolerated and improved the visual outcomes in patients with PCV as evaluated at 1-year follow-up examinations. However, there was a trend toward better vision improvement with fixed treatment every 2 months."
20	Ito 2022 [43]	Brolucizumab, 17	PCV (100%)	Japan	Retrospective Cohort	13 (86.7%)	77.8±2.7	0.28±0.05	12	IVA. Loading phase, 3-monthly IVBs. Maintenance phase, an IVB is every 12 weeks.	1. Patients enrolled from June 2020 to February 2021. 2. Confirmed PCV diagnosing.	BCVA, CMT, CCT, number of intravitreal injections, regression of polypoidal lesions evaluated by ICGA after 1 year, and complications.	"The IVBs appeared to be effective for improving both functional and anatomic outcomes in Japanese patients with PCV, with a high regression rate of polypoidal lesions."
21	Jeong 2016 [44]	Aflibercept, 29	Type 1 PCV, 16 (55.2%) Type 2 PCV, 13 (44.8%)	Japan	Prospective Cohort	11 (68.8%) 12 (92.3%)	69.7±7.4 64.2±6.6	0.76±0.41 0.62±0.30	6	IVA (2.0 mg). Loading phase, injection every month for the first 3 months Maintenance phase, one additional injection was administered at 4 months.	1. Patient enrolled from September 1, 2015, to March 31, 2016. 2. Confirmed PCV diagnosis.	Short-term efficacy of IVA of PCV percentage of polyp regression after 3-monthly injections.	"There was a difference in early treatment response with aflibercept between two subtypes of PCV. Type 1 polypoidal CNV showed better visual improvement with a higher percentage of polyp regression than type 2 IPCV."
22	Kawashima 2014 [45]	Aflibercept, 41	AMD, 15 (36.6%) PCV, 26 (63.4%)	Japan	Non-RCT	36 (87.9%)	75.6±8.0	0.40±0.37	6	IVA (2.0 mg). Loading phase, injections once a month for 3 months. Maintenance phase, administered bi-monthly.	1. Patient enrolled from December 1, 2012, to August 31, 2013. 2. With ranibizumab for longer than 6 months. 3. Showed recurrent or residual exudative changes after the last three injections.	Visual and anatomic outcomes in response to the conversion of treatment in PCV.	"In AMD or PCV patients refractory to ranibizumab, switching to aflibercept is generally effective regardless of patient genotype. PCV patients may benefit more significantly than AMD patients."
23	Kikushima 2017 [47]	Aflibercept, 146	AMD, 71 (50.7%) PCV, 69 (49.3%)	Japan	Retrospective Cohort	89 (60.9%)	75.9±8.1	0.45±0.39 0.36±0.35	12	IVA (2.0 mg/0.05 ml). Loading phase, 3-monthly injections.	1. Eyes with treatment-naïve AMD, including typical nAMD and PCV. 2. Baseline decimal BCVA ≤ 1.2 in the Landolt chart. 3. Minimum follow-up period of 12 months after the initial injection.	Efficacy and visual outcome and incidence of retreatment of AMD and PCV.	"These findings might be helpful for physicians when considering the optimal treatment regimen for exudative AMD."
24	Kikushima 2021 [46]	Aflibercept, 40	nAMD, 13 (32.5%) PCV, 27 (67.5%)	Japan	Retrospective Cohort	31 (77.5%)	71.8±8.0	0.12±0.09	60	IVA (2.0 mg/0.05 ml) Loading phase, 3-monthly injections.	1. Eyes with treatment-naïve AMD, including typical nAMD and PCV. 2. Baseline decimal BCVA ≥ 0.6 in the Landolt chart. 3. Minimum follow-up period of 60 months after the initial injection.	5-year visual and anatomic outcomes for patients secondary to exudative AMD.	"As-needed aflibercept monotherapy is a preferable treatment option for exudative AMD with good initial visual acuity regardless of nAMD or PCV during the 5-year study period."
25	Koizumi 2015 [48]	Aflibercept, 96	PCV (100%)	Japan	Retrospective Cohort	69 (78.4%)	72.3±7.4	0.31±0.32	3	IVA (2.0 mg). Loading phase, injection monthly for 3 months.	1. Patients enrolled from December 1, 2012, to October 31, 2013. 2. Confirmed PCV diagnosis.	Short-term outcomes of IVAs for PCV.	"IVAs for the treatment of a large number of PCV eyes were found to improve both visual acuity and macular morphology over the short term."
26	Kwon 2020 [49]	Aflibercept, 86	PCV (100%)	Korea	Retrospective Cohort	60 (69.8%)	65.6±7.26	0.23±0.09	12	IVA (2.0mg). Loading phase, injection monthly for 3 months.	1. Patients enrolled from January 2014 to December 2018. 2. Confirmed PCV diagnosis.	Efficacy of IVA in PCV.	"In cases of PCV with good visual acuity, intravitreal aflibercept injections decreased CSMT and were effective in maintaining visual acuity."
27	Lee 2016 [50]	Aflibercept, 40	PCV (100%)	Korea	Non-RCT	27 (67.5%)	67.0±9.2	NA	12	IVA (2.0 mg). Loading phase, injection monthly for 3 months. Maintenance, injection every 2 months.	1. Patients enrolled from September 2013 to July 2014. 2. Confirmed PCV diagnosis. 3. Polypoidal lesion located within 3,000 μm of the foveal center. 4. BCVA between 20/40 and 20/320. 5. evidence of intraretinal fluid, SRF, or sub-RPE fluid.	BCVA is defined as losing <15 ETDRS letters over the 12-month.	"Fixed-dosing aflibercept showed favorable outcomes in PCV patients at 12 months. However, some patients had worse outcomes because of fluid recurrence during maintenance dosing, and these patients would require additional treatments."
28	Lee 2019 [51]	Aflibercept, 40	PCV (100%)	Korea	Non-RCT	25 (62.5%)	69.5±8.6	61.5±11.1	14	IVA (2.0 mg). Loading phase, injection monthly for 3 months. Maintenance, injection every 2 months.	1. Patients enrolled from March 1, 2014, to September 31, 2016. 2. Patients older than 20 years. 3. BCVA letter score of 24 to 77 using ETDRS charts at a starting distance of 4 m. 4. Confirmed PCV diagnosing.	Efficacy of IVA for PCV without active polyps.	"Intravitreal afibercept improved the visual and anatomical outcomes of PCV with exudation from BVN after pre-treatment with PDT and/or anti-VEGF other than afibercept. Better vision, smaller lesion size, and absence of an inner retinal cyst after induction therapy may predict better visual outcome."
29	Maruyama-Inoue 2017 [52]	Aflibercept, 33	PCV (100%)	Japan	Retrospective Cohort	24 (72.7%)	70.2 ± 8.2	0.32±0.32	36	IVA (2.0 mg). Loading phase, injection monthly for 3 months and fixed dose. Maintenance, injection every 2 months OR PRN. Maintenance, as-needed basis.	1. Patients enrolled between March 2013 and May 2014 2. Confirmed PCV diagnosing.	Compare the 3-year follow-up results of IVA for the treatment of PCV and analyze factors influencing improvement in visual acuity.	"Intravitreal aflibercept was effective in improving the vision of patients with PCV, as evaluated at the 3-year follow-up. Fixed treatment might be an important factor influencing improvement in visual acuity."
30	Mishra 2022 [24]	Aflibercept, 8	PCV (100%)	USA	Retrospective Cohort	7 (87.5%)	62.3±7.7	0.70±0.36	96	IVA (1.25 mg/0.05 ml).	1. Patients enrolled from March 2017 and February 2018. 2. Confirmed PCV diagnosis.	Morphological outcomes of PCV undergoing IVZ monotherapy.	"IVZ leads to significant morphological changes on ICGA and OCT in terms of polyp regression and reduction of PED height, respectively, with a limited change in visual acuity. IVZ may serve as a cost-effective alternative to treat eyes with PCV."
31	Nishikawa 2019 [26]	Aflibercept, 98	Typical AMD, 32 PCV, 33	Japan	Non-RCT	55 (75.5%)	75.3±2.6	0.28±0.1	48	IVA (2.0 mg). Loading phase, injection monthly for 3 months. Maintenance, injection every 2 months.	1. Patients enrolled from December 2012 to December 2013. 2. Age older than 50 years, axial length less than 26.5 mm. 3. The presence of nAMD.	4-year visual outcome of aflibercept treatment in patients with AMD.	"The status of ELM, vitreoretinal adhesion, and choroidal thickness were predictive factors for final vision."
32	Nizawa 2021 [54]	Aflibercept, 37	PCV, 19 (51.4%) Non-PCV, 18 (48.6%)	Japan	Non-RCT	26 (70.3%)	73.5±7.6	0.35±0.23	12	IVA (2.0 mg/0.05 ml). Loading phase, every 4 weeks for the first 3 months. Maintenance phase, injection every 8 weeks for 12 months.	1. Between September 2016 and March 2019. 2. Age ≥50 years. 3. Treatment-naive with nAMD with PCV or non-PCV. 4. The presence of SRF or SRH. 5. BCVA ≥ 0.1 (logMAR).	BCVA and CFT	"IVA administered by a fixed dosing regimen led to significant improvements of the central MS, BCVA, and macular morphology at 1 year in eyes with nAMD with or without PCV. These results were not significantly different between eyes with non-PCV and with PCV. The improvements of the MS of the retina of the central 2° in a subgroup whose BCVA remained unchanged through the 12-month experimental period were also significant. We conclude that the MS of the central 2° might be a better marker than the BCVA in determining the effectiveness of IVA treatments and might be helpful in determining early effects on the retina before BCVA changes can be detected."
33	Ogasawara 2018 [55]	Aflibercept, 109	PCV, 64 (58.7%) Typical AMD, 45 (41.3%)	Japan	Retrospective Cohort	85 (79.4%)	74.9±9.0	0. 47±0.32	12	IVA (2.0 mg). Loading phase, injection monthly for 3 months. Maintenance, injection every 2 months.	1. Patients age 50 years or older. 2. More than 12 months of follow-up. 3. Imaging data available from each visit.	Visual outcomes in eyes treated with IVA for AMD or PCV.	"Although poorer BCVA and the presence of SRF predicted larger gains in BCVA in both subtypes treated with aflibercept, eyes with typical nAMD had greater improvement if no PED was present, while eyes with PCV had greater improvement if CVH was present."
34	Pengyi 2022 [62]	Aflibercept, 73	2 mg aflibercept, 38 (52.1%) 4 mg aflibercept, 35 (47.9%)	China	Non-RCT	41 (56.2%)	66±9.3 63.67±10.9	0.69±0.27 0.68±0.30	6	IVA (2.0 mg/0.05 ml) or (4 mg /0.1 ml) 3 + PRN regimen once a month.	1. Age > 50 years. 2. Patients who meet the diagnostic criteria for PCV. 3. Patients with active CNV and serous PED, with PED height > 100 µm. 4. Patients who had not received intravitreal drug injections in the past 1 month.	Effectiveness and safety of IVA for PCV resistant to ranibizumab.	"Both 2 mg and 4 mg aflibercept can effectively treat ranibizumab-resistant PCV with serous PED, and improve the anatomical structure of the retina and BCVA. In addition, 4 mg aflibercept can accelerate the recovery of PED and CMT."
35	Rouvas 2021 [56]	Aflibercept, 30	TAE, 14 (46.7%) PRN, 16 (53.3%)	Greece	Non-RCT	6 (43%) 7 (44%)	69.40±4.20 67.10±3.70	0.57±0.24 0.65±0.18	12	IVA (2.0 mg). Loading phase, injection monthly for 3 months. Maintenance, injection every 2 months.	1. Patients enrolled from 2015 through 2018. 2. Confirmed PCV diagnosis.	1-year outcomes of TAE and PRN treatment regimens with aflibercept for PCV.	"We highlighted the superiority of TAE regime with IVAs, which seems to yield better functional outcomes by preventing recurrence and subfoveal fibrosis, although a greater number of injections is required."
36	Saito 2015 [57]	Aflibercept, 46	PCV (100%)	Japan	Retrospective Cohort	51 (78.5%)	75.7±5.8	NA	6	IVA (2 mg/0.05 ml) Maintenance, bimonthly.	1. Patients enrolled from December 2012 to August 2013. 2. Confirmed PCV diagnosis.	Changes in subfoveal thickness after switching to IVA for PCV.	"The choroidal thickness in PCV eyes significantly decreased after switching to intravitreal aflibercept injection. Aflibercept may help prevent CVH near or under the RPE, which might help achieve greater occlusion of polypoidal lesions compared with ranibizumab."
37	Sayanagi 2024 [58]	Aflibercept, 81	PCV (100%)	Japan	Retrospective Chart Review	57 (70%)	73.7±6.9	0.29±0.36	12	IVA (2 mg/0.05 ml). Loading dose, 3 initial loading doses followed by monthly injections.	1. Patients enrolled from January 2013, with at least 12 months of follow-up since starting treatment. 2. Confirmed PCV diagnosis. 3. No patients had been treated previously for PCV.	Effect of polyp regression and reduction on treatment efficacy in PCV.	"Regarding IVA therapy for PCV, the presence or absence of complete polyp regression at the end of the loading phase affected the treatment outcome, whereas the degree of polyp reduction in cases of residual polyps had no effect."
38	Tamachi 2020 [59]	Aflibercept, 62	PCV (100%)	Japan	Retrospective Chart Review	35 (68.6%)	74.9±7.3	0.24±0.32	12	IVA (2 mg/0.05 ml). Loading dose, 3 initial loading doses at 5-week intervals.	1. Patients enrolled from January 2013 and August 2014. 2. Confirmed PCV diagnosis. 3. Patients aged over 50 years.	1-year outcomes of IVA using a TAE regimen for PCV.	"IVA using a TAE regimen improved visual and anatomical outcomes in eyes with PCV at 1 year using a protocol to adjust the injection intervals specifically for each patient so as to obtain no retinal exudation."
39	Wan Nam 2022 [53]	Brolucizumab, 26	PCV (100%)	Korea	Retrospective Chart Review	15 (57.7%)	69.0±7.6	0.31±0.28	4.27	IVB (6.0 mg) followed by PRN retreatment monthly.	1. Patients enrolled between April 2021 and April 2022. 2. Confirmed PCV diagnosis. 3. Patients aged over 50.	Efficacy and safety of brolucizumab in the treatment of refractory serous PED secondary to PCV.	"Intravitreal brolucizumab may be an effective and safe treatment option for refractory serous PEDs in patients with PCV."
40	Wolff 2018 [60]	Aflibercept, 34	PCV (100%)	France	Non-RCT	21 (61.8%)	72.3±1.7	NA	6	IVA (2.0 mg). Loading phase, injection monthly for 3 months. Maintenance, injection every 2 months.	1. Patients enrolled between April 2021 and April 2022. 2. Confirmed PCV diagnosis. 3. Caucasian patients >45 years old.	Evaluation of BCVA at the 6-month visit.	"Aflibercept as a monotherapy provided both a significant visual gain and the regression of polypoidal dilations. Aflibercept use in monotherapy may contribute to reducing the hemorrhagic risk and atrophy linked to PDT."
41	Y. Hsu 2022 [23]	Brolucizumab, 10	wAMD or PCV	Korea	Retrospective Cohort	NA	67.6	NA		IVB	1. Confirmed PCV diagnosis. 2. Patients who had follow-ups at 1 week and 5 weeks.	Short-term effects of brolucizumab in the treatment of wAMD or polypoidal choroidopathy.	"Treatment with brolucizumab resulted in anatomical improvement for all patients with persistent SRF. Limited efficacy was seen for persistent PED. Brolucizumab appears to be a safe and effective option for treatment-resistant SRF. Future multicenter collaborative studies are warranted."
42	Yamamoto 2015 [61]	Aflibercept, 90	PCV (100%)	Japan	Retrospective Cohort	68 (78.2%)	71.1±7.3	0.31±0.32	12	IVA (2.0 mg). Loading phase, injection monthly for 3 months. Maintenance, injection every 2 months.	1. Patients enrolled from December 1, 2012, to October 31, 2014. 2. Confirmed PCV diagnosis.	Visual and morphologic outcomes of IVA for treatment-naïve PCV.	"Intravitreal aflibercept administered over 1 year improved both visual acuity and macular morphology in a large number of treatment-naïve eyes with PCV."
43	Yeom 2023 [27]	Brolucizumab, 81	PCV, 37 (45.7%)	Korea	Retrospective Cohort	55 (67.9%)	70.6±6.6	NA	12	IVB	1. Patients enrolled from July 2021 to December 2022. 2. Confirmed PCV diagnosis. 3. Aged ≥ 50 years.	Efficacy and safety of IVB as a switch therapy.	"Identified structural biomarkers may predict treatment response and select an appropriate therapeutic strategy."
44	Yoneyama 2020 [28]	Aflibercept, 234	PCV, 116 (49.6%) Typical nAMD, 118 (50.4%)	Japan	Retrospective Cohort	170 (72.7%)	74.9±8.2	0.42±0.37	12	IVA (2 mg/0.05 ml). Loading phase, injection monthly for 3 months.	1. Patients enrolled from January 2013 and 2018. 2. The patients with exudative AMD, including PCV and typical nAMD. 3. Completed 12 months of follow-up.	Visual outcomes and the need for additional injections after the initial 3-monthly IVA.	"The variants of ARMS2 and CFH are informative for both physicians and patients to predict recurrence and to quantify the need for additional injections."

Quality Assessment

Our included randomized controlled trails was judged low risk in all ROB1 domains (Table 2). Regarding our included cohort studies, 79% were of fair quality, scoring between 8 and 10, while 21% had good quality (Table 3). Concerning case series studies, 66% were of good quality, while the rest were of fair quality (Table 4). As to our non-randomized studies, 55% judged good quality, whereas 45 % were of fair quality according to the MINORS Criteria (Table 5).

**Table 2:** The Cochrane Collaboration’s tool for assessing the risk of bias of randomized controlled trials

Study ID	Random sequence generation (selection bias)	Allocation concealment (selection bias)	Blinding of participants and personnel (performance bias)	Blinding of outcome assessment (Detection bias)	Incomplete outcome data (attrition bias)	Selective reporting (reporting bias)	Other Bias	
	Low\High\Unclear risk of bias	Low\High\Unclear risk of bias	Low\High\Unclear risk of bias	Low\High\Unclear risk of bias	Low\High\Unclear risk of bias	Low\High\Unclear risk of bias	Low\High\Unclear risk of bias	
								Ogura 2021 [13]	Low	Low	Low	Low	Low	Low	Low	

**Table 3:** NIH quality assessment tool for observational cohort studies Quality rating: good (11-14 points), fair (7.5-10.5 points), poor (0-7 points) Yes = 1; No = 0.5;  NR, NA, CD = 0 CD: Cannot determine; NR: Not reported, NA: Not applicable, NIH: National Institutes of Health

ID	1. Was the research question or objective in this paper clearly stated?	2. Were eligibility/selection criteria for the study population prespecified and clearly described?	3. Were the participants in the study representative of those who would be eligible for the test/service/intervention in the general or clinical population of interest?	4. Were all eligible participants that met the prespecified entry criteria enrolled?	5. Was the sample size sufficiently large to provide confidence in the findings?	6. For the analyses in this paper, were the exposure(s) of interest measured prior to the outcome(s) being measured?	7. Was the time frame sufficient so that one could reasonably expect to see an association between exposure and outcome if it existed?	8. For exposures that can vary in amount or level, did the study examine different levels of the exposure as related to the outcome (eg, categories of exposure, or exposure measured as continuous variable)?	9. Were the exposure measures (independent variables) clearly defined,valid, reliable, and implemented consistently across all study participants?	10. Was the exposure(s) assessed more than once over time?	11. Were the outcome measures prespecified, clearly defined, valid, reliable, and assessed consistently across all study participants?	12. Were the people assessing the outcomes blinded to the participants' exposures/interventions?	13. Was the loss to follow-up after baseline 20% or less? Were those lost to follow-up accounted for in the analysis?	14. Were key potential confounding variables measured and adjusted statistically for their impact on the relationship between exposure(s) and outcome(s)?	Total scores	Quality rating
Cho 2023 [32]	Y	Y	Y	Y	N	Y	Y	NA	Y	N	Y	NA	Y	NR	10	Fair
Fukuda 2021 [35]	Y	Y	Y	Y	N	Y	NA	NA	Y	N	Y	NA	Y	Y	10	Fair
Fukuda 2023 [36]	CD	Y	Y	Y	N	Y	Y	NA	Y	N	Y	NA	Y	Y	10	Fair
Azuma 2016 [30]	CD	Y	Y	Y	N	Y	Y	NA	Y	N	Y	NA	NR	NR	8	Fair
Chakraborty 2022 [31]	CD	Y	Y	Y	N	Y	Y	NA	Y	N	Y	NA	Y	NR	9	Fair
Daizumoto 2016 [21]	Y	y	y	y	N	Y	Y	NA	Y	N	Y	NA	Y	Y	11	Good
Fukuda 2020 [22]	Y	Y	Y	Y	N	Y	Y	NA	Y	N	Y	NA	NR	Y	10	Fair
Hara 2016 [37]	CD	Y	Y	Y	N	Y	Y	NA	Y	N	Y	NA	N	Y	9.5	Fair
Hoshino 2024 [38]	Y	Y	Y	Y	N	Y	Y	NA	Y	N	Y	NA	Y	NR	10	Fair
Hosokawa 2015 [40]	Y	Y	Y	Y	N	Y	Y	NA	Y	Y	Y	NA	NR	Y	10.5	Fair
Ito 2022 [43]	Y	Y	Y	Y	N	Y	Y	NA	Y	N	Y	NA	Y	NR	10	Fair
Kikushima 2017 [47]	Y	Y	Y	Y	N	Y	Y	NA	Y	Y	Y	NA	Y	Y	11.5	Good
Kikushima 2021 [46]	Y	Y	Y	Y	N	Y	Y	NA	Y	Y	Y	NA	NR	Y	10.5	Fair
Koizumi 2015 [48]	Y	Y	Y	Y	N	Y	Y	NA	Y	N	Y	NA	Y	Y	11	Good
Kwon 2020 [49]	CD	Y	Y	Y	N	Y	Y	NA	Y	Y	Y	NA	Y	Y	10.5	Fair
Maruyama-Inoue 2017 [52]	Y	Y	Y	Y	N	Y	Y	NA	Y	N	Y	NA	NR	Y	10	Fair
Mishra 2022 [24]	Y	Y	Y	Y	N	Y	Y	NA	Y	N	Y	NA	Y	NR	10	Fair
Ogasawara 2018 [55]	CD	Y	Y	Y	N	Y	Y	NA	Y	Y	Y	NA	Y	Y	10.5	Fair
Saito 2015 [57]	Y	Y	Y	Y	N	Y	Y	NA	Y	Y	Y	NA	Y	NR	10.5	Fair
Y. Hsu 2022 [23]	Y	Y	Y	Y	N	Y	NA	NA	Y	N	Y	NA	Y	NR	9	Fair
Yamamoto 2015 [61]	Y	Y	Y	Y	N	Y	Y	NA	Y	Y	Y	NA	Y	Y	11.5	Good
Yeom 2023 [27]	Y	Y	Y	Y	N	Y	Y	NA	Y	N	Y	NA	Y	NR	10	Fair
Yoneyama 2020 [28]	Y	Y	Y	Y	N	Y	Y	NA	Y	N	Y	NA	Y	Y	11	Good
Chi Moon 2015 [25]	Y	N	Y	Y	N	Y	Y	NA	Y	N	Y	NA	Y	NR	9.5	Fair
Dabir 2023 [20]	Y	Y	Y	Y	N	Y	Y	NA	Y	N	Y	NA	Y	NR	10	Fair
Sayanagi 2024 [58]	Y	Y	Y	Y	N	Y	Y	NA	Y	N	Y	NA	Y	NR	10	Fair
Tamachi 2020 [59]	Y	Y	Y	Y	N	Y	Y	NA	Y	Y	Y	NA	Y	Y	11.5	Good
Wan Nam 2022 [53]	CD	Y	Y	Y	N	Y	Y	NA	Y	Y	Y	NA	Y	NR	9.5	Fair
Jeong 2016 [44]	Y	Y	Y	Y	N	Y	Y	NA	Y	Y	Y	NA	Y	NR	10.5	Fair

**Table 4:** NIH quality assessment tool for case series studies Quality rating: good (7.5-9 points), fair (5-7 points), poor (4.5-0 points) CD: Cannot determine; NR: Not reported; NA: Not applicable

ID	1. Was the study question or objective clearly stated?	2. Was the study population clearly and fully described, including a case definition?	3. Were the cases consecutive?	4. Were the subjects comparable?	5. Was the intervention clearly described?	6. Were the outcome measures clearly defined, valid, reliable, and implemented consistently across all study participants?	7. Was the length of follow-up adequate?	8. Were the statistical methods well-described?	9. Were the results well-described?	Total scores: (Yes = 1; No= 0.5; NR, NA, CD = 0)	Quality rating
Cho 2023 [32]	Y	Y	Y	Y	Y	Y	Y	Y	Y	9	Good
Hosokawa 2015 [40]	N	Y	Y	CD	Y	Y	Y	Y	Y	7.5	Good
Ijiri 2015 [41]	N	Y	Y	CD	Y	Y	Y	N	Y	6.5	Fair

**Table 5:** MINORS Criteria for assessment of non-randomized clinical trials Quality rating: good (5.5-7 points) or fair 4-5.5 points) or poor (3.5-0 points) ) Yes: 1; No: 0.5;  NR, NA, CD: 0 CD: Cannot determine; NR: Not reported; NA: Not applicable; MINORS: Methodological Index for Non-Randomized Studies

ID	1. A stated aim of the study?	2. Inclusion of consecutive patients?	3. Prospective collection of data?	4. Endpoint appropriate to the study aim?	5. Unbiased evaluation of endpoints?	6. Follow-up period appropriate to the major endpoint?	7. Loss to follow up not exceeding 5%?	Total scores	Quality rating
Arakawa 2017 [29]	Y	Y	Y	Y	N	Y	Y	6.5	Good
Farooq 2017 [34]	Y	Y	N	Y	N	Y	Y	6	Good
Inoue 2015 [42]	Y	Y	N	Y	N	Y	Y	6	Good
Kawashima 2014 [45]	Y	Y	N	Y	N	Y	Y	6	Good
Lee 2019 [51]	Y	Y	Y	Y	N	Y	Y	6.5	Good
Nishikawa 2019 [26]	Y	Y	N	Y	N	Y	Y	6	Good
Nizawa 2021 [54]	Y	Y	N	Y	N	Y	N	5.5	Good
Pengyi 2022 [62]	Y	Y	N	Y	N	Y	Y	6	Good
Rouvas 2021 [56]	Y	Y	N	Y	N	Y	Y	6	Good
Wolff 2018 [60]	Y	Y	Y	Y	N	Y	Y	6.5	Good
Lee 2016 [50]	Y	Y	Y	Y	N	Y	N	6	Good

BCVA, logMAR

**Figure 2:**
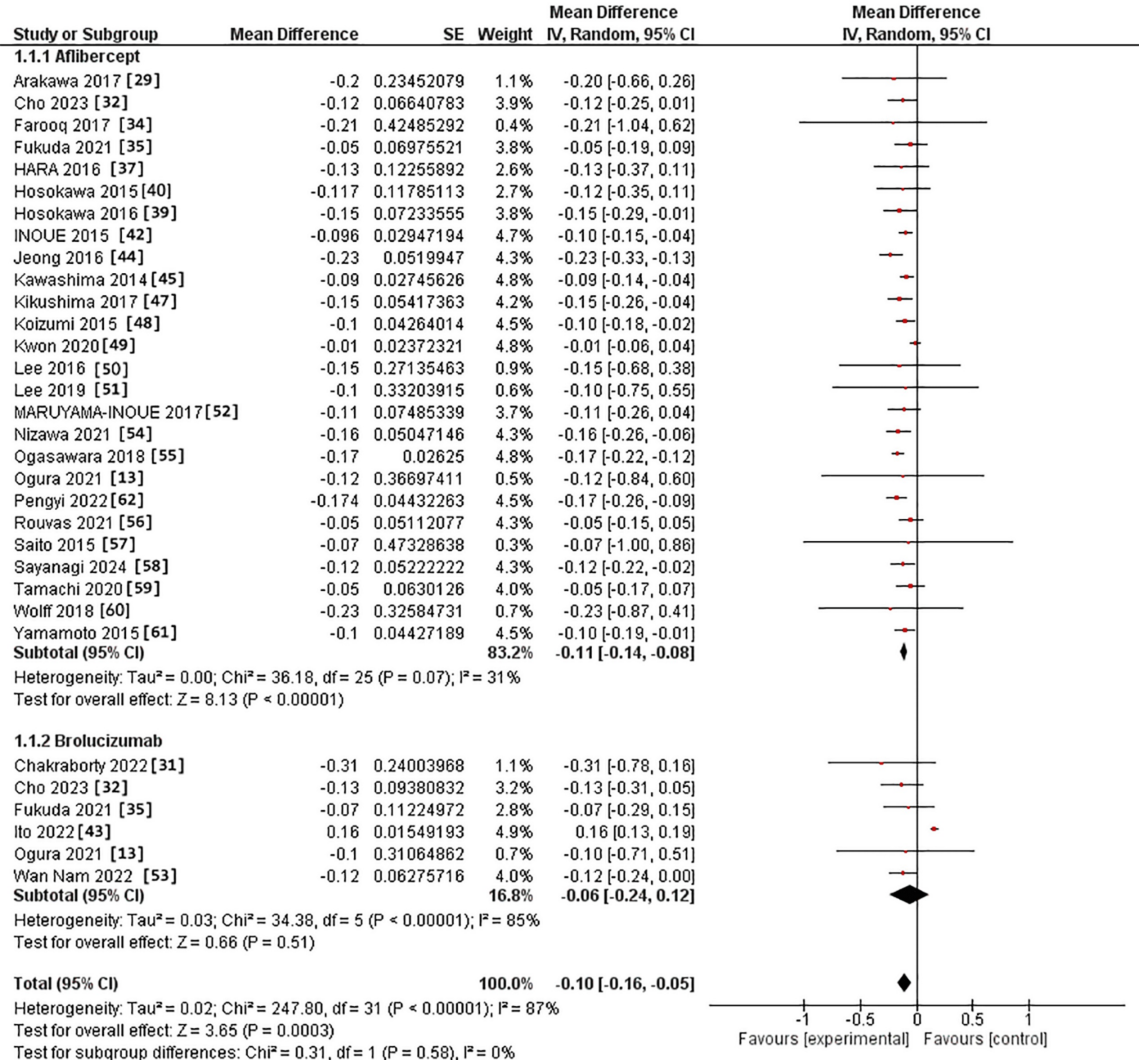
Forest plot of BCVA in the 3-6 month subgroup BCVA: Best corrected visual acuity

In the 3-6-month subgroup, BCVA was assessed in 26 studies in IVA arm [13,29,32,34,37,39,40,42,44-52,54-62]. It was evaluated in six studies in IVB arm [13,31,32,35,43,53]. The MD for BCVA after IVA use was -0.11 versus -0.06 in the IVB arm. However, the two interventions had no substantial difference (p = 0.58). The pooled studies exhibited heterogeneity in both intervention groups, with Chi^2^ p-values and I^2^ statistics indicating heterogeneity (0.07 and 31%) for the IVA group and (>0.0001 and 85%) for the IVB group, respectively (Figure 2).

**Figure 3:**
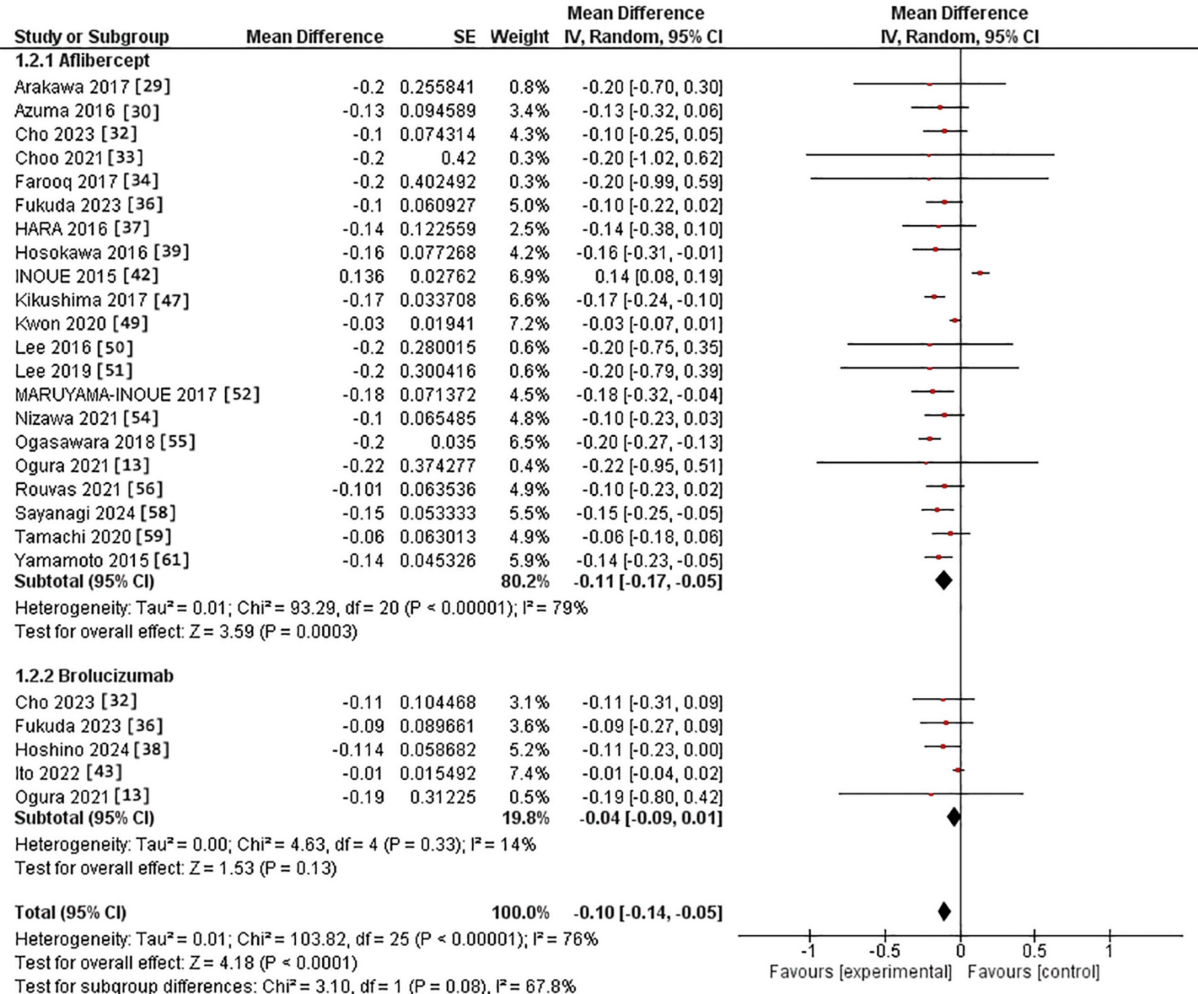
Forest plot of BCVA in the 12 months assessment BCVA: Best corrected visual acuity

Regarding the 12-month assessment, 21 studies were in the IVA arm [13,29,30,32-34,36,37,39,42,47,49-52,54-56,58,59,61]. Meanwhile, five studies were in the IVB arm [13,32,36,38,43]. The MD for BCVA after IVA use was -0.11 versus -0.04 in the IVB arm. However, the two interventions had no substantial difference (p = 0.08). The pooled studies were heterogeneous in the IVA group, with Chi^2^-p and I^2^ being (>0.0001 and 79%). In comparison, they were homogeneous in the IVB group, with Chi^2^-p and I^2^ being (0.31 and 17%, respectively) (Figure 3).

Polypoidal Regression

**Figure 4:**
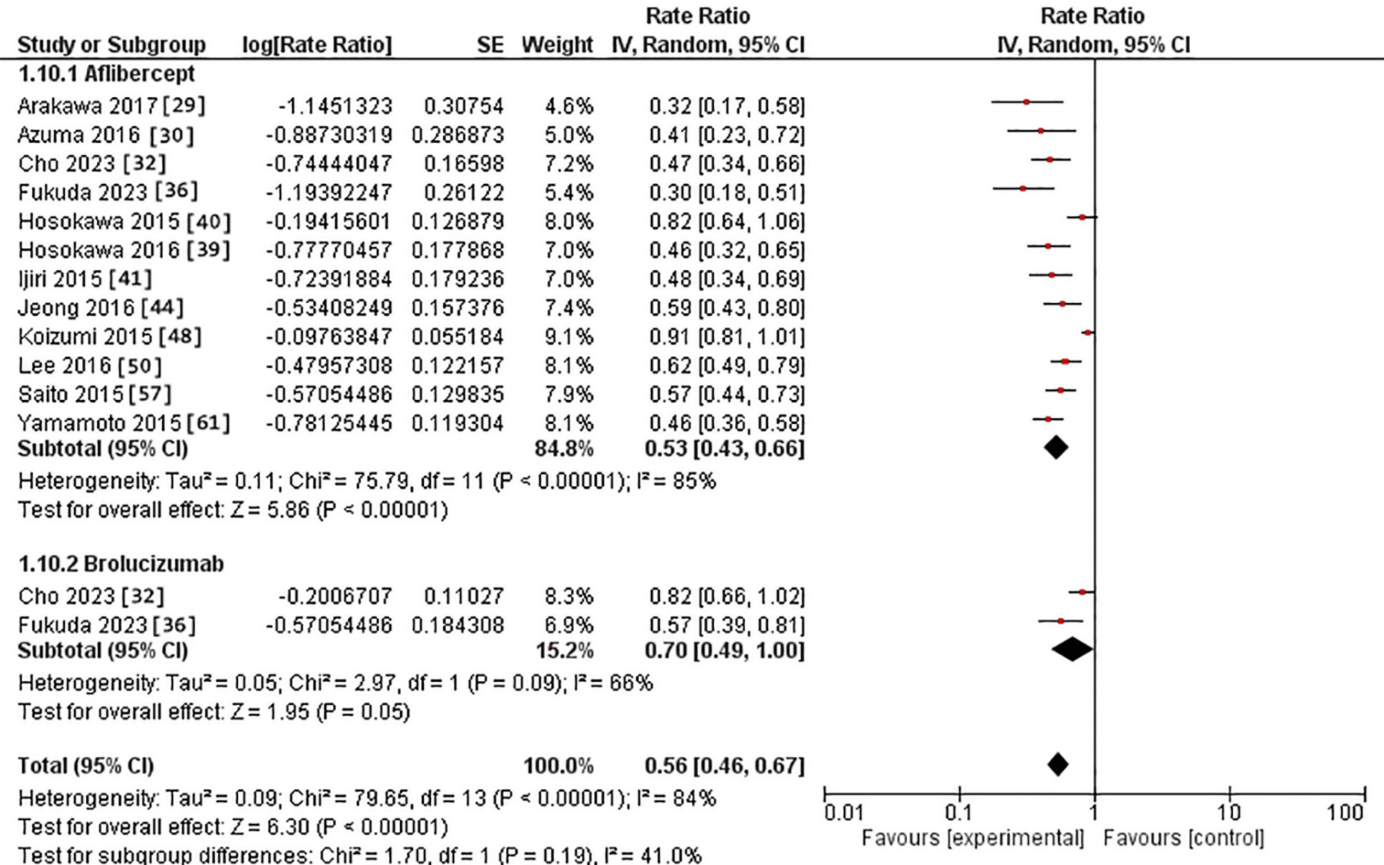
Forest plot of polypoidal regression in the 3-6 month subgroup

At 3-6-month subgroup, polypoidal regression was assessed in 12 studies in the IVA arm [29,30,32,36,39-41,44,48,50,57,61]. It was evaluated in two studies in the IVB arm [32,36]. The RR for polypoidal regression after IVA use was 53% versus 70% in the IVB arm. However, the two interventions had no substantial difference (p = 0.19). The pooled studies exhibited heterogeneity in both intervention groups, with Chi^2^ p-values and I^2^ statistics indicating heterogeneity (>0.0001 and 85%) for the IVA group and (0.09 and 66%) for the IVB group, respectively (Figure 4).

**Figure 5:**
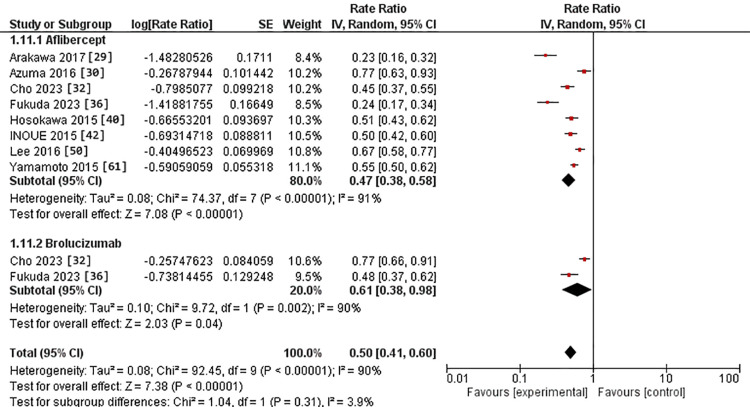
Forest plot of polypoidal regression in the 12-month assessment

Regarding the 12-month assessment, eight studies were in the IVA arm [29,30,32,36,40,42,50,61]. Two studies were in the IVB arm [32,36]. The RR for polypoidal regression after IVA use was 47% versus 61% in the IVB arm. However, there was no substantial difference between the two interventions (p = 0.31). The pooled studies exhibited heterogeneity in both intervention groups, with Chi^2^ p-values and I^2^ statistics indicating heterogeneity (>0.0001 and 91%) for the IVA group and (0.002 and 90%) for the IVB group, respectively (Figure 5).

CRT

**Figure 6:**
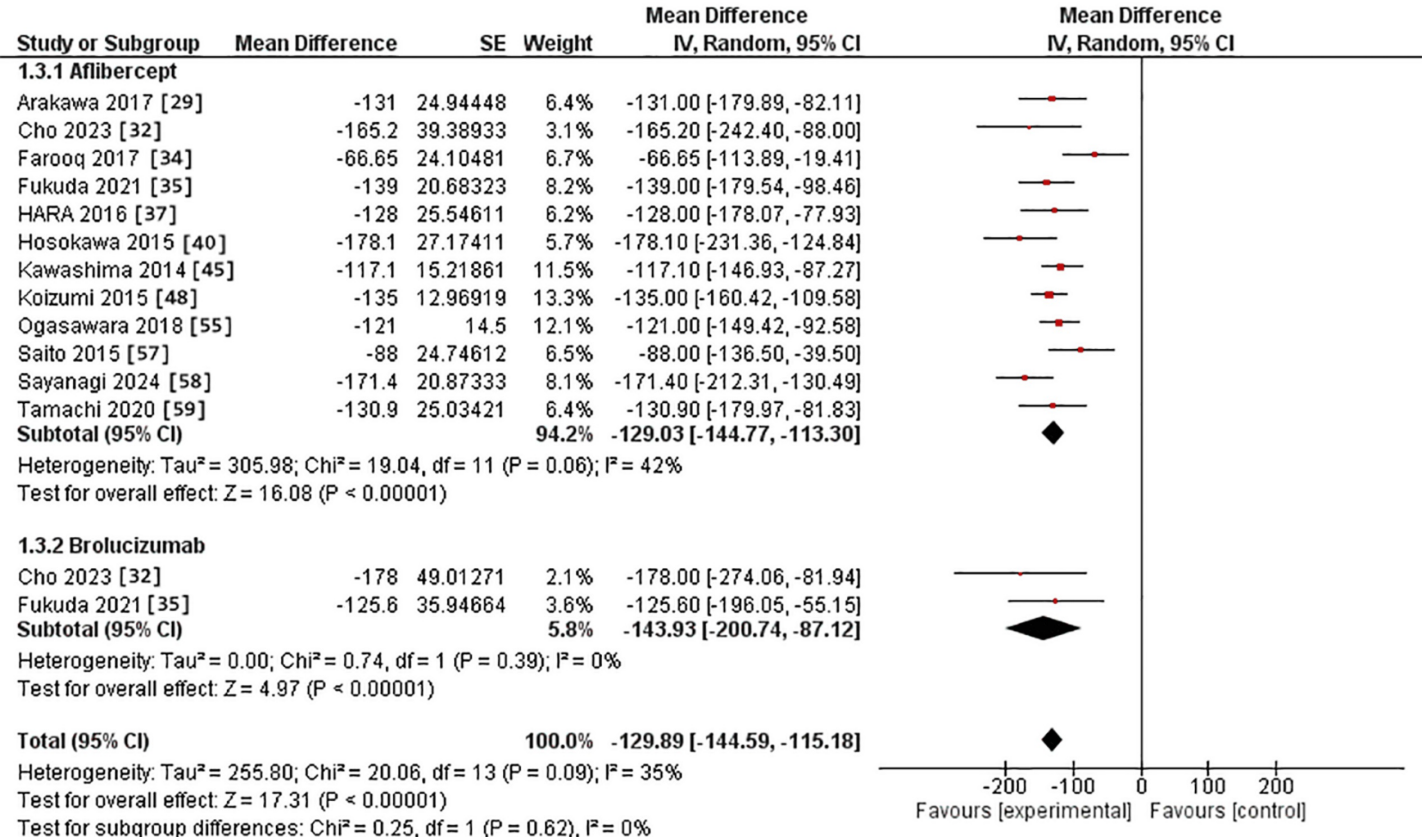
Forest plot of CRT in the 3-6 month subgroup CRT: Central retinal thickness

At 3-6-month subgroup, CRT was assessed in 12 studies in the IVA arm [29,32,34,35,37,40,45,48,55,57-59]. It was assessed in two studies in the IVB arm [32,35]. The MD for CRT after IVA use was -129.03 versus -143.93 in the IVB arm. However, there was no substantial difference between the two interventions (p = 0.62). The pooled studies were heterogeneous in the IVA group, with Chi^2^-p and I^2^ being (0.06 and 42%). In comparison, they were homogeneous in the IVB group, with Chi^2^-p and I^2^ being (0.39 and 0%, respectively) (Figure 6).

**Figure 7:**
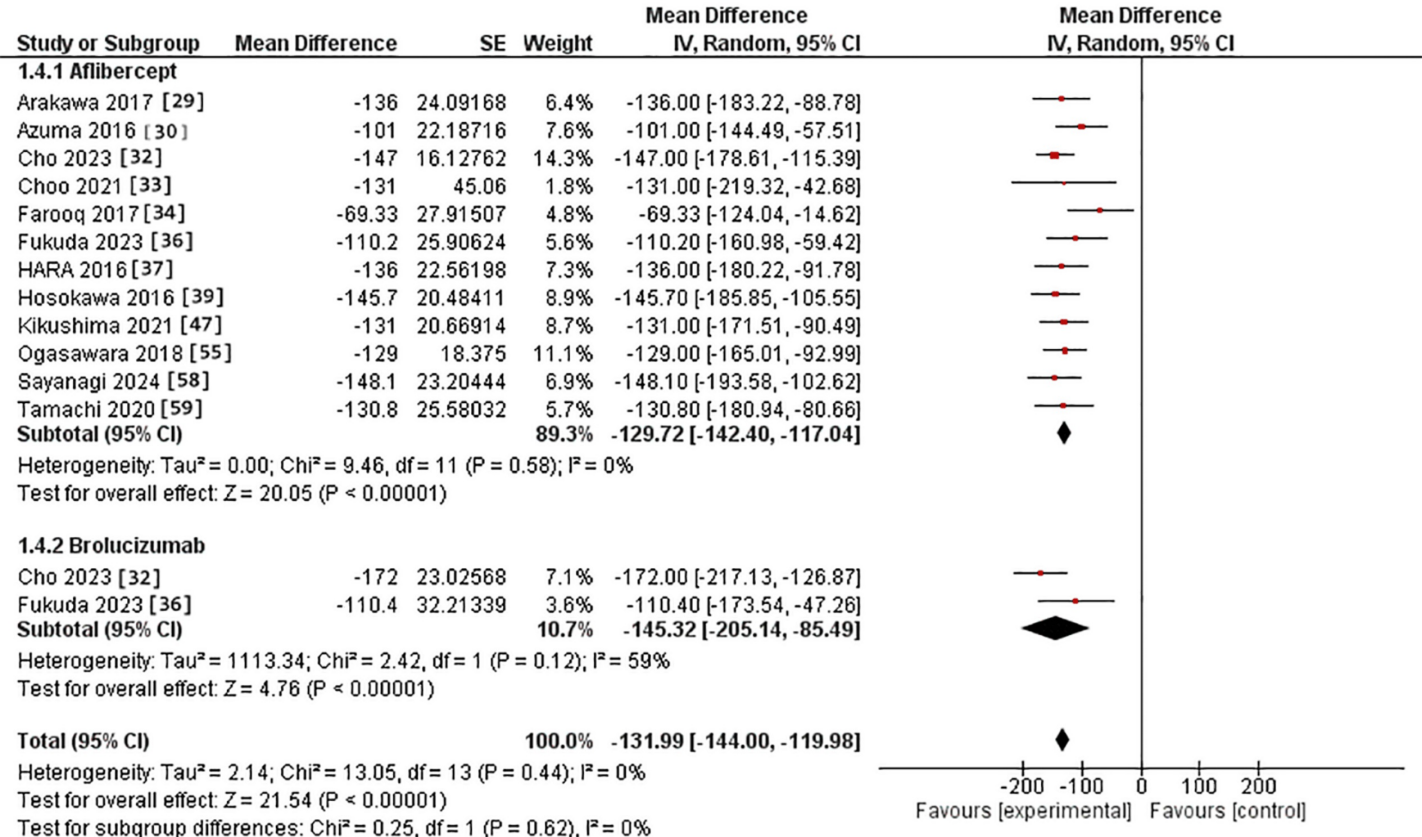
Forest plot of CRT in the 12-month assessment CRT: Central retinal thickness

Regarding the 12-month assessment, 12 studies were in the IVA arm [29,30,32-34,36,37,39,46,55,58,59]. Two studies were in the IVB arm [32,36]. The MD for CRT after IVA use was -129.72 versus -145.32 in the IVB arm. However, there was no significant difference between the two interventions (p = 0.62). The pooled studies were homogeneous in both intervention groups, with Chi^2^ p-values and I^2^ statistics (0.58 and 0%) for the IVA group and (0.12 and 59%) for the IVB group (Figure 7).

CCT

**Figure 8:**
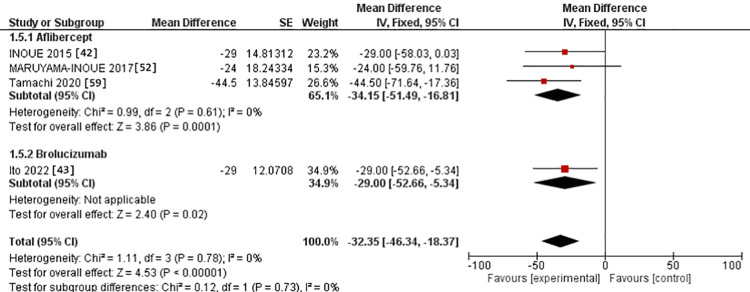
Forest plot of CCT in the 3-6-month subgroup CCT: Central choroidal thickness

In the 3-6-month subgroup, CCT was assessed in three studies in the IVA arm [42,52,59]. It was studied in one study in the IVB arm [43]. The MD for CCT after IVA use was -34.15 versus -29.00 in the IVB arm. However, there was no significant difference between the two interventions (p = 0.73). The studies were homogenous in the IVA group, with Chi^2^-p and I^2^ being (0.61 and 0%, respectively) (Figure 8).

**Figure 9:**
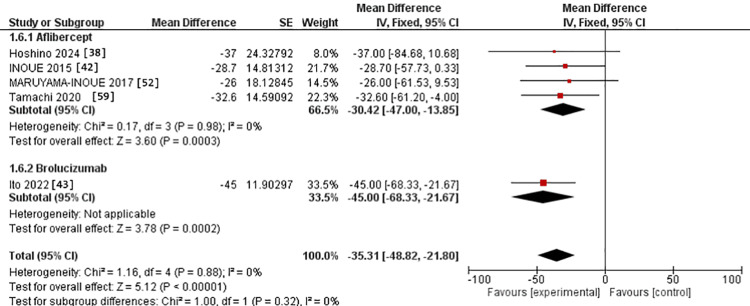
Forest plot of CCT in the 12-month assessment CCT: Central choroidal thickness

Regarding the 12-month assessment, four studies were in the IVA arm [38,43,52,59]. Only one study was in the IVB arm [43]. The MD for CCT after IVA use was -30.42 versus -45.00 in the IVB arm. However, there was no substantial difference between the two interventions (p = 0.32). The studies were homogeneous in the IVA group, with Chi^2^-p and I^2^ being (0.98 and 0%, respectively) (Figure 9).

Central Macular Thickness

**Figure 10:**
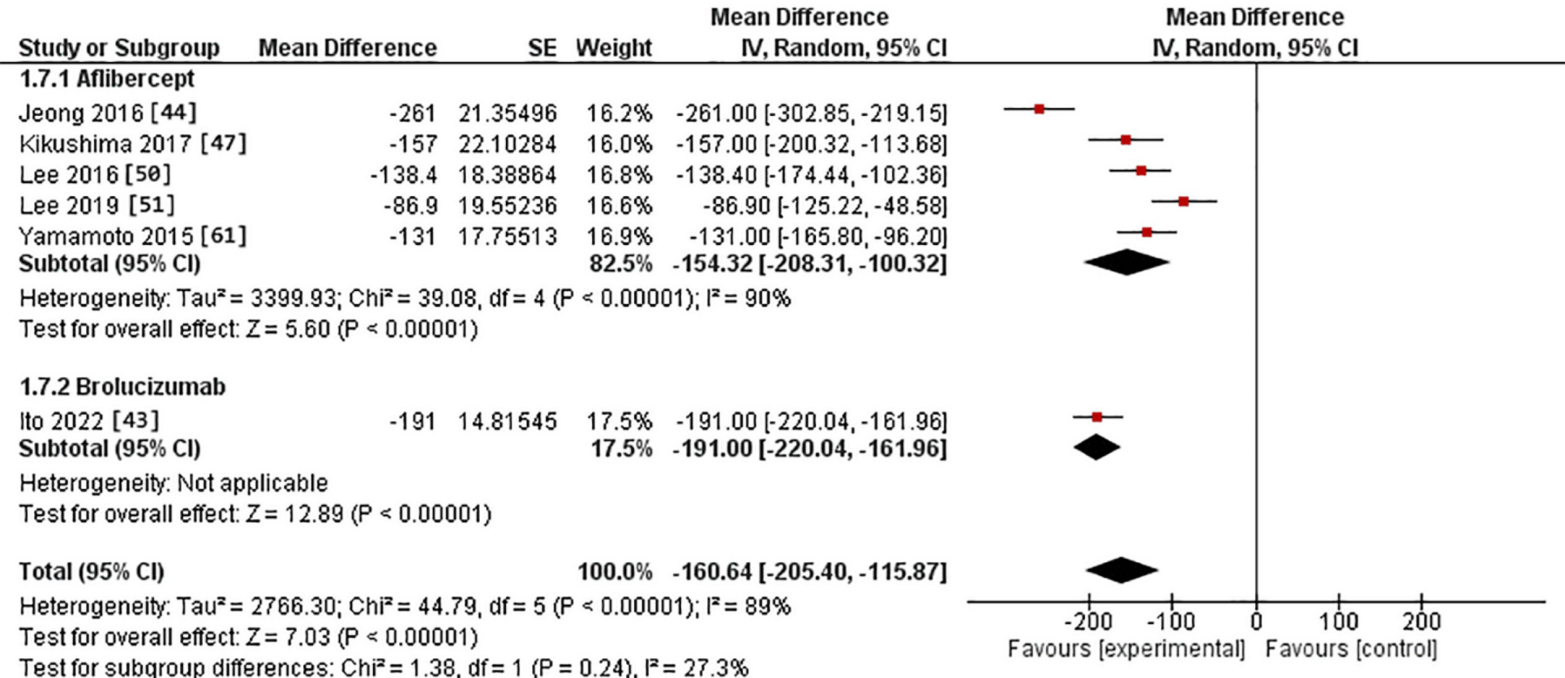
Forest plot of the central macular thickness in the 3-6-month subgroup

In the 3-6-month subgroup, central macular thickness was assessed in five studies in the IVA arm [44,47,50,51,61]. It was evaluated in one study in the IVB arm [43]. The MD for central macular thickness after IVA use was -154.32 versus -191.00 in the IVB arm. However, there was no substantial difference between the two interventions (p = 0.24). The pooled studies were heterogeneous in the IVA group, with Chi^2^-p and I^2^ being (>0.0001 and 90%, respectively) (Figure 10).

**Figure 11:**
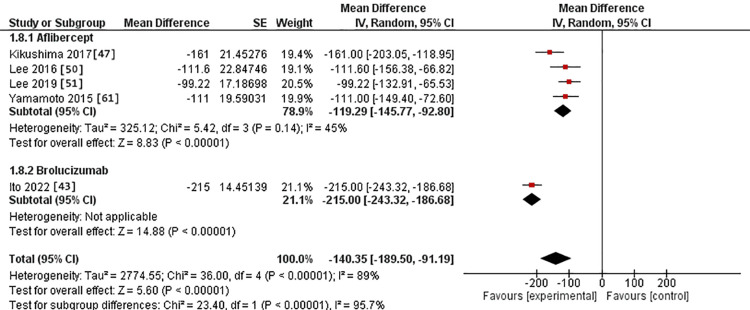
Forest plot of the central macular thickness in the 12-month assessment

Regarding the 12-month assessment, four studies were conducted in the IVA arm [48,51,52,62]. One was conducted in the IVB arm [43]. The MD for central macular thickness after IVA use was -119.29 versus -215.00 in the IVB arm. This difference was statistically substantial, favoring IVB over IVA (p > 0.0001). The studies were homogeneous in the IVA group, with Chi^2^-p value and I^2^ being (0.14 and 45%, respectively) (Figure 11).

CFT

**Figure 12:**
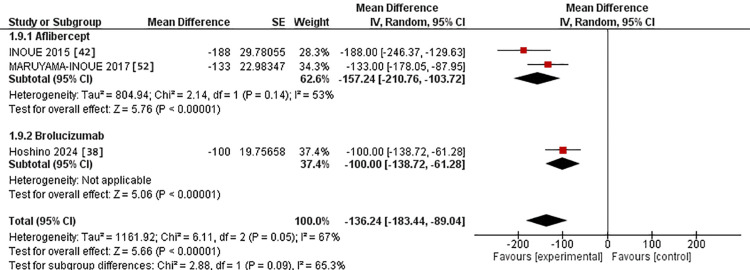
Forest plot of CFT in the 12-month assessment CFT: Central foveal thickness

Regarding the 12-month assessment, two studies were in the IVA arm [42,52] versus one study in the IVB arm [38]. The MD for CFT after IVA use was -157.24 versus -100.00 in the IVB arm. However, there was no substantial difference between the two interventions (p = 0.09). The studies were homogeneous in the IVA group, with Chi^2^-p value and I^2^ being (0.14 and 53%, respectively) (Figure 12).

Discussion

Our meta-analysis retrieved 35 studies to compare between IVA and IVB in PCV patients. BCVA, polypoidal regression, CRT, CCT, and CFT showed no significant differences between the drugs at the two studied time points. Notably, at 12 months, IVB demonstrated a statistically significant advantage over IVA in central macular thickness. Nevertheless, the findings suggest comparable efficacy between IVA and IVB in managing PCV. IVB stands out as the smallest available anti-VEGF [11,22]. Due to its high solubility, IVB can be concentrated to 120 mg/mL, allowing administration of 0.06 mg IVB per each 0.05 mL injection. In a solitary intravitreal injection, the quantity of IVB molecules and their affinity for VEGF is estimated to be about 11-fold higher compared to IVA [11,22]. This characteristic may confer several advantages in terms of morphological and angiographic outcomes when treating PCV. 

In the treatment regimen for PCV, the primary objective is the resolution of exudation coupled with the regression of polypoidal lesions. Achieving regression of polypoidal lesions is particularly crucial as it is anticipated to prolong the interval to recurrence [63]. As reported in previous studies [61,63-66], the complete regression rate of polypoidal lesions following IVAs ranged between 45.8% and 56.7%. Conversely, when combining therapy involving PDT and anti-VEGF agents, the complete regression rate was between 70% and 87%. In the study conducted by Fukuda et al., the complete regression rate of polypoidal lesions one month after three-monthly IVA administrations was reported to be 30.3%, indicating a lower rate compared to findings from previous studies [36]. Previous studies have reported complete regression rates of polypoidal lesions ranging from 78.6% to 82.0% one month after three-monthly injections of IVB, and from 73.9% to 93.1% at one year following the initial injection [35,43,64,67]. These findings serve as a reference for assessing the efficacy of IVB in treating PCV. In the study by Fukuda et al., the complete regression rate was reported to be 56.5% at both the 3-month and 12-month visits, which is lower compared to findings from previous studies [36]. This variance in the complete regression rates could be attributed to differences in the cohorts' conditions, including variations in ICGA findings and discrepancies in treatment regimens across the studies [36]. 

Furthermore, it is worth noting that the number of additional injections administered was smaller in the IVB-treated group compared to the IVA-treated group. This discrepancy in the number of additional injections could be attributed to differences in the complete regression rate of polypoidal lesions and VEGF binding affinity between the two drugs.

Interestingly, a recent study reveals that IVA demonstrates greater binding affinity to VEGF compared to IVB and ranibizumab, as evidenced in both in vivo and in vitro studies. While binding affinity is a critical factor influencing the efficacy of anti-VEGF agents, other factors may also contribute to differences in treatment outcomes, such as choroidal thickness and macular thickness. For instance, molecular size and penetration depth of the anti-VEGF agents into retinal and choroidal layers can affect their therapeutic impact. IVB, being a smaller molecule, may exhibit superior penetration into the sub-RPE region, potentially leading to more pronounced reductions in macular thickness.

Additionally, the pharmacokinetics and half-life of these drugs in the vitreous humor may influence the duration of VEGF suppression, which could affect the number of injections required and the consistency of therapeutic effects over time. Differences in the inflammatory response or immune modulation triggered by the drugs may also play a role, as some anti-VEGF agents have been associated with varying levels of ocular inflammation. Lastly, patient-specific factors, such as baseline choroidal thickness, underlying systemic conditions, or prior treatments, could further modulate individual responses to therapy [68]. Further investigations are warranted to explore whether the binding affinity to VEGF correlates with the resolution of exudation in PCV. Irrespective of the treatment regimen and modality employed, the management of PCV typically entails prolonged and persistent use of anti-VEGF drugs. Consequently, these findings hold significant implications for both patients and healthcare providers. Consistent utilization of IVB may lead to anticipated reductions in medication costs and injection frequency.

Despite the more potent VEGF suppression achieved by IVB compared to IVA, morphological changes such as CRT and subfoveal choroidal thickness did not exhibit significant differences between the two treatment groups [36]. Notably, there is a lack of studies comparing choroidal thickness changes following administering these two drugs in eyes with exudative AMD. 

In the investigation conducted by Cho et al., the average BCVA subsequent to the initial loading injection demonstrated enhancement by 0.11 logMAR unit in the group treated with IVB, and by 0.10 logMAR unit in the group treated with IVA [32]. Their visual outcomes were similar to those observed in the HAWK study, which investigated nAMD. In the HAWK study, the group treated with IVB 6.0 mg gained 6.6 letters, while the group treated with IVA 2.0 mg gained 6.8 letters after 48 weeks of treatment. Moreover, concerning CRT, observations from the HAWK trial demonstrated a significant reduction in central subfield thickness within the IVB-treated cohort compared to the IVA-treated cohort at the 48-week mark [69]. Likewise, the investigation involving a cohort exclusively diagnosed with PCV noted that the reduction in CRT was more pronounced in the IVB-treated group compared to the IVA-treated group [32].

Previous investigations have suggested that IVA might result in a more pronounced decrease in choroidal thickness compared to other anti-VEGF agents, such as bevacizumab or ranibizumab [70,71]. The research conducted by Cho et al. noted a substantial decrease in choroidal thickness subsequent to IVB therapy compared to IVA therapy. This finding aligns with the hypothesis that IVB’s smaller molecular size and higher concentration may enhance its penetration into the choroid, potentially leading to more pronounced effects on choroidal thickness [32]. Considering that the variation in choroidal thickness reduction among anti-VEGF agents is more pronounced in patients with PCV compared to other types of macular neovascularization, it is imperative to validate reductions in foveal thickness following IVB therapy across various types of nAMD in future studies [70]. The change in choroidal thickness is of importance as individuals with thinner choroids are at an increased risk of developing macular atrophy [72,73]. Furthermore, prior indications suggest that decreased choroidal thickness may be linked to suppressed disease activity in eyes afflicted by PCV [74]. Further investigations are warranted to explore the clinical significance of changes in choroidal thickness associated with IVB treatment. Long-term follow-ups extending beyond 24 months would provide valuable insights into the efficacy and safety profile of IVB therapy in managing various retinal conditions.

The underlying reasons for the observed variances in anatomical outcomes, such as polyp regression, between the groups treated with IVB and IVA, remain uncertain. Additional investigations are warranted to clarify the contributing factors underlying these differences. The observed variance could potentially be associated with the notably higher molar concentration of the anti-VEGF agent in IVB. Specifically, the molar concentration in IVB surpasses that in IVA and ranibizumab by approximately 11 and 22 times, respectively [75]. Another hypothesis is that this outcome may be linked to the enhanced penetration of IVB into the sub-RPE region. Preclinical investigations have demonstrated that IVB exhibits robust penetration into the retina, reaching the RPE/choroid with greater efficacy compared to full-sized immunoglobulin G [11,76]. Future studies should focus on elucidating the precise differences and therapeutic mechanisms of action for PCV treatment between IVB and other anti-VEGF agents. These investigations will provide valuable insights into optimizing treatment strategies for PCV patients. 

The strengths of our study include being the pioneering meta-analysis to compare the efficacy of IVB versus IVA, which provided valuable insights into their relative performance. Additionally, our meticulous stratification of follow-up groups into two distinct categories enhances the precision of our findings through these periods. However, certain limitations must be acknowledged. Firstly, our analysis relied on indirect comparisons, potentially introducing bias and limiting the direct applicability of our results. This could attribute our findings to potential biases stemming from differences in study design, population characteristics, or measurement methods between the compared datasets. Secondly, the inclusion of mixed study designs, primarily retrospective, may impact our conclusions' robustness. Lastly, the relatively small population of IVB compared to IVA may affect the generalizability of our findings and warrant further investigation. Our study highlights the need for more direct head-to-head trials comparing IVB and IVA, particularly in larger and more diverse patient populations. Such trials would provide more robust evidence to guide treatment decisions and optimize patient outcomes in the management of PCV.

## Conclusions

Our meta-analysis comparing IVA and IVB in PCV revealed no substantial differences in clinical parameters, including BCVA, polypoidal regression, CRT, CCT, and CFT at 6 or 12 months. Notably, IVB exhibited an advantage over IVA in central macular thickness at 12 months. However, overall, both drugs demonstrated comparable efficacy in managing PCV While our findings provide valuable insights, our limitations underscore the importance of conducting direct head-to-head trials with larger, more diverse patient populations. 

## References

[1] Goldhardt R, Rosen BS (2019). Polypoidal choroidal vasculopathy. Curr Ophthalmol Rep.

[2] Koh AH, Chen LJ, Chen SJ (2013). Polypoidal choroidal vasculopathy: evidence-based guidelines for clinical diagnosis and treatment. Retina.

[3] Wong CW, Yanagi Y, Lee WK, Ogura Y, Yeo I, Wong TY, Cheung CM (2016). Age-related macular degeneration and polypoidal choroidal vasculopathy in Asians. Prog Retin Eye Res.

[4] Maruko I, Iida T, Saito M, Nagayama D, Saito K (2007). Clinical characteristics of exudative age-related macular degeneration in Japanese patients. Am J Ophthalmol.

[5] Lorentzen TD, Subhi Y, Sørensen TL (2018). Prevalence of polypoidal choroidal vasculopathy in white patients with exudative age-related macular degeneration: systematic review and meta-analysis. Retina.

[6] Lee WK, Iida T, Ogura Y (2018). Efficacy and safety of intravitreal aflibercept for polypoidal choroidal vasculopathy in the PLANET study: a randomized clinical trial. JAMA Ophthalmol.

[7] Pereira FB, Veloso CE, Kokame GT, Nehemy MB (2015). Characteristics of neovascular age-related macular degeneration in Brazilian patients. Ophthalmologica.

[8] Kokame GT, deCarlo TE, Kaneko KN, Omizo JN, Lian R (2019). Anti-vascular endothelial growth factor resistance in exudative macular degeneration and polypoidal choroidal vasculopathy. Ophthalmol Retina.

[9] Cheung CM, Lai TY, Ruamviboonsuk P (2018). Polypoidal choroidal vasculopathy: definition, pathogenesis, diagnosis, and management. Ophthalmology.

[10] Teo KY, Gillies M, Fraser-Bell S (2018). The use of vascular endothelial growth factor inhibitors and complementary treatment options in polypoidal choroidal vasculopathy: a subtype of neovascular age-related macular degeneration. Int J Mol Sci.

[11] Nguyen QD, Das A, Do DV (2020). Brolucizumab: evolution through preclinical and clinical studies and the implications for the management of neovascular age-related macular degeneration. Ophthalmology.

[12] Dugel PU, Singh RP, Koh A (2021). HAWK and HARRIER: ninety-six-week outcomes from the phase 3 trials of brolucizumab for neovascular age-related macular degeneration. Ophthalmology.

[13] Ogura Y, Jaffe GJ, Cheung CM (2022). Efficacy and safety of brolucizumab versus aflibercept in eyes with polypoidal choroidal vasculopathy in Japanese participants of HAWK. Br J Ophthalmol.

[14] Matsumoto H, Hoshino J, Mukai R, Nakamura K, Akiyama H (2021). Short-term outcomes of intravitreal brolucizumab for treatment-naïve neovascular age-related macular degeneration with type 1 choroidal neovascularization including polypoidal choroidal vasculopathy. Sci Rep.

[15] (2019). Cochrane handbook for systematic reviews of interventions. Cochrane Handbook for Systematic Reviews of Interventions. 2nd Edition..

[16] Page MJ, McKenzie JE, Bossuyt PM (2021). The PRISMA 2020 statement: an updated guideline for reporting systematic reviews. BMJ.

[17] Higgins JP, Altman DG, Gøtzsche PC (2011). The Cochrane Collaboration's tool for assessing risk of bias in randomised trials. BMJ.

[18] Institution NH. https://www.nhlbi.nih (2024). Study quality assessment tools. https://www.nhlbi.nih.gov/health-topics/study-quality-assessment-tools.

[19] Slim K, Nini E, Forestier D, Kwiatkowski F, Panis Y, Chipponi J (2003). Methodological index for non-randomized studies (MINORS): development and validation of a new instrument. ANZ J Surg.

[20] Dabir S, Mohankumar A, Khatri MG, Rajan M (2024). Brolucizumab in age-related macular neovascularization (BRAIN study): efficacy, optical coherence tomography biomarkers, and safety profile. Indian J Ophthalmol.

[21] Daizumoto E, Mitamura Y, Sano H (2017). Changes of choroidal structure after intravitreal aflibercept therapy for polypoidal choroidal vasculopathy. Br J Ophthalmol.

[22] Fukuda Y, Sakurada Y, Sugiyama A (2020). Pachydrusen in fellow eyes predict response to aflibercept monotherapy in patients with polypoidal choroidal vasculopathy. J Clin Med.

[23] Hsu AY, Lin CY, Lin CJ (2022). Short-term effects of brolucizumab in the treatment of wet age-related macular degeneration or polypoidal choroidopathy refractory to previous anti-vascular endothelial growth factor therapy. Medicina (Kaunas).

[24] Mishra SB, Singh SR, Goyal P, Chakurkar R, Govindhari V, Goud A, Chhablani J (2021). Regression in polypoidal choroidal vasculopathy treated with ziv-aflibercept monotherapy - short term study. Saudi J Ophthalmol.

[25] Moon DR, Lee DK, Kim SH, You YS, Kwon OW (2015). Aflibercept treatment for neovascular age-related macular degeneration and polypoidal choroidal vasculopathy refractory to anti-vascular endothelial growth factor. Korean J Ophthalmol.

[26] Nishikawa K, Oishi A, Hata M (2019). Four-year outcome of aflibercept for neovascular age-related macular degeneration and polypoidal choroidal vasculopathy. Sci Rep.

[27] Yeom H, Kwon HJ, Kim YJ, Lee J, Yoon YH, Lee JY (2023). Real-world study to evaluate the efficacy and safety of intravitreal brolucizumab for refractory neovascular age-related macular degeneration. Sci Rep.

[28] Yoneyama S, Sakurada Y, Kikushima W (2020). Genetic factors associated with response to as-needed aflibercept therapy for typical neovascular age-related macular degeneration and polypoidal choroidal vasculopathy. Sci Rep.

[29] Arakawa A, Inoue M, Sato S, Yamane S, Kadonosono K (2017). Efficacy of intravitreal aflibercept injections for Japanese patients with polypoidal choroidal vasculopathy. Clin Ophthalmol.

[30] Azuma K, Obata R, Nomura Y, Tan X, Takahashi H, Yanagi Y (2016). ngiographic findings of ranibizumab-resistant polypoidal choroidal vasculopathy after switching to a treat-and-extend regimen with intravitreal aflibercept. Retina.

[31] Chakraborty D, Maiti A, Sengupta S, Mondal S, Nandi K, Chakraborty S (2022). Initial experience in treating polypoidal choroidal vasculopathy with brolucizumab in Indian eyes - A multicenter retrospective study. Indian J Ophthalmol.

[32] Cho HJ, Kang KH, Yoon W, Lee J, Kim CG, Kim JW (2023). Intravitreal brolucizumab and aflibercept for polypoidal choroidal vasculopathy. J Ocul Pharmacol Ther.

[33] Choo HG, Lee JH, Oh HS, Kim SH, You YS, Kwon OW (2021). One-year outcomes of fixed-dosing aflibercept therapy for pre treated and naive polypoidal choroidal vasculopathy patient. BMC Ophthalmol.

[34] Farooq A, Frazier H, Marcus WB, Fechter C, Singh H, Marcus DM (2017). Intravitreal aflibercept for neovascular polypoidal choroidal vasculopathy in a predominantly non-Asian population: RIVAL results. Ophthalmic Surg Lasers Imaging Retina.

[35] Fukuda Y, Sakurada Y, Matsubara M, Hasebe Y, Sugiyama A, Kikushima W, Kashiwagi K (2021). Comparison of outcomes between three monthly brolucizumab and aflibercept injections for polypoidal choroidal vasculopathy. Biomedicines.

[36] Fukuda Y, Sakurada Y, Matsubara M, Kotoda Y, Kasai Y, Sugiyama A, Kashiwagi K (2023). Comparison of one-year outcomes between as-needed brolucizumab and aflibercept for polypoidal choroidal vasculopathy. Jpn J Ophthalmol.

[37] Hara C, Sawa M, Sayanagi K, Nishida K (2016). One-year results of intravitreal aflibercept for polypoidal choroidal vasculopathy. Retina.

[38] Hoshino J, Matsumoto H, Nakamura K, Akiyama H (2024). Predicting treatment outcomes of intravitreal brolucizumab for polypoidal choroidal vasculopathy through noninvasive assessment of polypoidal lesion blood flow with optical coherence tomography angiography. Sci Rep.

[39] Hosokawa M, Morizane Y, Hirano M (2017). One-year outcomes of a treat-and-extend regimen of intravitreal aflibercept for polypoidal choroidal vasculopathy. Jpn J Ophthalmol.

[40] Hosokawa M, Shiraga F, Yamashita A (2015). Six-month results of intravitreal aflibercept injections for patients with polypoidal choroidal vasculopathy. Br J Ophthalmol.

[41] Ijiri S, Sugiyama K (2015). Short-term efficacy of intravitreal aflibercept for patients with treatment-naïve polypoidal choroidal vasculopathy. Graefes Arch Clin Exp Ophthalmol.

[42] Inoue M, Yamane S, Taoka R, Arakawa A, Kadonosono K (2016). Aflibercept for polypoidal choroidal vasculopathy: as needed versus fixed interval dosing. Retina.

[43] Ito A, Maruyama-Inoue M, Kitajima Y, Ikeda S, Inoue T, Kadonosono K (2022). One-year outcomes of intravitreal brolucizumab injections in patients with polypoidal choroidal vasculopathy. Sci Rep.

[44] Jeong S, Sagong M (2017). Short-term efficacy of intravitreal aflibercept depending on angiographic classification of polypoidal choroidal vasculopathy. Br J Ophthalmol.

[45] Kawashima Y, Oishi A, Tsujikawa A (2015). Effects of aflibercept for ranibizumab-resistant neovascular age-related macular degeneration and polypoidal choroidal vasculopathy. Graefes Arch Clin Exp Ophthalmol.

[46] Kikushima W, Sakurada Y, Sugiyama A, Yoneyama S, Matsubara M, Fukuda Y, Kashiwagi K (2021). Five-year outcome of aflibercept monotherapy for exudative age-related macular degeneration with good baseline visual acuity. J Clin Med.

[47] Kikushima W, Sakurada Y, Yoneyama S (2017). Incidence and risk factors of retreatment after three-monthly aflibercept therapy for exudative age-related macular degeneration. Sci Rep.

[48] Koizumi H, Kano M, Yamamoto A (2015). Aflibercept therapy for polypoidal choroidal vasculopathy: short-term results of a multicentre study. Br J Ophthalmol.

[49] Kwon JM, Pak KY, Lee JJ, Sagong M, Kim HW (2021). One-year results of aflibercept treatment for polypoidal choroidal vasculopathy with good visual acuity. Korean J Ophthalmol.

[50] Lee JE, Shin JP, Kim HW (2017). Efficacy of fixed-dosing aflibercept for treating polypoidal choroidal vasculopathy: 1-year results of the VAULT study. Graefes Arch Clin Exp Ophthalmol.

[51] Lee SE, Jang JW, Kang SW, Park KH, Lee DW, Kim JH, Bae K (2019). Intravitreal aflibercept for active polypoidal choroidal vasculopathy without active polyps. Sci Rep.

[52] Maruyama-Inoue M, Sato S, Yamane S, Kadonosono K (2018). Intravitreal injection of aflibercept in patients with polypoidal choroidal vasculopathy: a 3-year follow-up. Retina.

[53] Nam SW, Byun Z, Ham DI, Kong M (2022). Response to brolucizumab treatment for refractory serous pigment epithelial detachment secondary to polypoidal choroidal vasculopathy. BMC Ophthalmol.

[54] Nizawa T, Kitahashi M, Baba T (2021). Improvements of retinal sensitivity after intravitreal injection of aflibercept in eyes with neovascular age-related macular degeneration with or without polypoidal choroidal vasculopathy. Ophthalmologica.

[55] Ogasawara M, Koizumi H, Yamamoto A (2018). Prognostic factors after aflibercept therapy for typical age-related macular degeneration and polypoidal choroidal vasculopathy. Jpn J Ophthalmol.

[56] Rouvas A, Gouliopoulos N, Douvali M (2021). One year outcomes of treat and extend and pro re nata (PRN) treatment regimens with aflibercept for polypoidal choroidal vasculopathy. Eur J Ophthalmol.

[57] Saito M, Kano M, Itagaki K, Ise S, Imaizumi K, Sekiryu T (2016). Subfoveal choroidal thickness in polypoidal choroidal vasculopathy after switching to intravitreal aflibercept injection. Jpn J Ophthalmol.

[58] Sayanagi K, Fujimoto S, Hara C (2024). Effect of polyp regression and reduction on treatment efficacy in polypoidal choroidal vasculopathy treated with aflibercept. Sci Rep.

[59] Tamachi T, Kohno T, Yamamoto M (2020). One-year results of a treat-and-extend regimen of intravitreal aflibercept for polypoidal choroidal vasculopathy. Ophthalmol Ther.

[60] Wolff B, Vasseur V, Cahuzac A (2018). Aflibercept treatment in polypoidal choroidal vasculopathy: results of a prospective study in a Caucasian population. Ophthalmologica.

[61] Yamamoto A, Okada AA, Kano M (2015). One-year results of intravitreal aflibercept for polypoidal choroidal vasculopathy. Ophthalmology.

[62] Zhou P, Yang L, Xu Y (2022). Effect and safety of aflibercept in the treatment of polypoidal choroidal vasculopathy with ranibizumab-resistant serous pigment epithelial detachment. Chin J Exp Ophthalmol.

[63] Morimoto M, Matsumoto H, Mimura K, Akiyama H (2017). Two-year results of a treat-and-extend regimen with aflibercept for polypoidal choroidal vasculopathy. Graefes Arch Clin Exp Ophthalmol.

[64] Matsumiya W, Honda S, Otsuka K (2017). One-year outcome of combination therapy with intravitreal aflibercept and verteporfin photodynamic therapy for polypoidal choroidal vasculopathy. Graefes Arch Clin Exp Ophthalmol.

[65] Kikushima W, Sakurada Y, Sugiyama A (2017). Comparison of two-year outcomes after photodynamic therapy with ranibizumab or aflibercept for polypoidal choroidal vasculopathy. Sci Rep.

[66] Kikushima W, Sakurada Y, Sugiyama A, Tanabe N, Kume A, Iijima H (2017). Comparison of initial treatment between 3-monthly intravitreal aflibercept monotherapy and combined photodynamic therapy with single intravitreal aflibercept for polypoidal choroidal vasculopathy. Graefes Arch Clin Exp Ophthalmol.

[67] Tanaka K, Koizumi H, Tamashiro T (2022). Short-term results for brolucizumab in treatment-naïve neovascular age-related macular degeneration: a Japanese multicenter study. Jpn J Ophthalmol.

[68] Schubert W, Terjung C, Rafique A, Romano C, Ellinger P, Rittenhouse KD (2022). Evaluation of molecular properties versus in vivo performance of aflibercept, brolucizumab, and ranibizumab in a retinal vascular hyperpermeability model. Transl Vis Sci Technol.

[69] Dugel PU, Koh A, Ogura Y (2020). HAWK and HARRIER: phase 3, multicenter, randomized, double-masked trials of brolucizumab for neovascular age-related macular degeneration. Ophthalmology.

[70] Kim JH, Lee TG, Chang YS, Kim CG, Cho SW (2016). Short-term choroidal thickness changes in patients treated with either ranibizumab or aflibercept: a comparative study. Br J Ophthalmol.

[71] Gharbiya M, Cruciani F, Mariotti C, Grandinetti F, Marenco M, Cacace V (2015). Choroidal thickness changes after intravitreal anti-vascular endothelial growth factor therapy for age-related macular degeneration: ranibizumab versus aflibercept. J Ocul Pharmacol Ther.

[72] Sadda SR, Abdelfattah NS, Lei J (2020). Spectral-domain OCT analysis of risk factors for macular atrophy development in the HARBOR study for neovascular age-related macular degeneration. Ophthalmology.

[73] Cho HJ, Yoo SG, Kim HS, Kim JH, Kim CG, Lee TG, Kim JW (2015). Risk factors for geographic atrophy after intravitreal ranibizumab injections for retinal angiomatous proliferation. Am J Ophthalmol.

[74] Koizumi H, Kano M, Yamamoto A (2016). Subfoveal choroidal thickness during aflibercept therapy for neovascular age-related macular degeneration: twelve-month results. Ophthalmology.

[75] Holz FG, Dugel PU, Weissgerber G (2016). Single-chain antibody fragment VEGF inhibitor RTH258 for neovascular age-related macular degeneration: a randomized controlled study. Ophthalmology.

[76] Borras L, Gunde T, Tietz J, Bauer U, Hulmann-Cottier V, Grimshaw JP, Urech DM (2010). Generic approach for the generation of stable humanized single-chain Fv fragments from rabbit monoclonal antibodies. J Biol Chem.

